# Stellate cells are in utero markers of pancreatic disease in cystic fibrosis

**DOI:** 10.1186/s10020-024-00871-2

**Published:** 2024-08-07

**Authors:** Shih-Hsing Leir, Svyatoslav Tkachenko, Alekh Paranjapye, Frederick Meckler, Arnaud J. Van Wettere, Jenny L. Kerschner, Elizabeth Kuznetsov, Makayla Schacht, Pulak Gillurkar, Misha Regouski, Iuri Viotti Perisse, Cheyenne M. Marriott, Ying Liu, Ian Bunderson, Kenneth L. White, Irina A. Polejaeva, Ann Harris

**Affiliations:** 1 Department of Genetics and Genome Sciences, Case Western Reserve University School of Medicine, Cleveland, OH 44106-4955 USA; 2 Department of Animal, Dairy and Veterinary Sciences, Utah State University, Logan, UT USA; 3 Present Address: Department of Genetics, University of Pennsylvania, Philadelphia, PA USA

**Keywords:** Pancreatic disease, Cystic fibrosis (CF), CFTR, In utero development, Stellate cells, Single-cell RNA-seq

## Abstract

**Background:**

Pancreatic fibrosis is an early diagnostic feature of the common inherited disorder cystic fibrosis (CF). Many people with CF (pwCF) are pancreatic insufficient from birth and the replacement of acinar tissue with cystic lesions and fibrosis is a progressive phenotype that may later lead to diabetes. Little is known about the initiating events in the fibrotic process though it may be a sequela of inflammation in the pancreatic ducts resulting from loss of CFTR impairing normal fluid secretion. Here we use a sheep model of CF (*CFTR*^*−/−*^) to examine the evolution of pancreatic disease through gestation.

**Methods:**

Fetal pancreas was collected at six time points from 50-days of gestation through to term, which is equivalent to ~ 13 weeks to term in human. RNA was extracted from tissue for bulk RNA-seq and single cells were prepared from 80-day, 120-day and term samples for scRNA-seq. Data were validated by immunochemistry.

**Results:**

Transcriptomic evidence from bulk RNA-seq showed alterations in the *CFTR*^*−/−*^ pancreas by 65-days of gestation, which are accompanied by marked pathological changes by 80-days of gestation. These include a fibrotic response, confirmed by immunostaining for COL1A1, αSMA and SPARC, together with acinar loss. Moreover, using scRNA-seq we identify a unique cell population that is significantly overrepresented in the *CFTR*^*−/−*^ animals at 80- and 120-days gestation, as are stellate cells at term.

**Conclusion:**

The transcriptomic changes and cellular imbalance that we observe likely have pivotal roles in the evolution of CF pancreatic disease and may provide therapeutic opportunities to delay or prevent pancreatic destruction in CF.

**Supplementary Information:**

The online version contains supplementary material available at 10.1186/s10020-024-00871-2.

## Introduction

The first description of the common inherited disorder cystic fibrosis (CF) noted the profound alterations in pancreatic tissue which were reflected in the original name “cystic fibrosis of the pancreas” (Andersen 1938; Andersen 1958). CF pancreatic disease has a prenatal origin (Reid et al. 1997; Boue et al. 1986) and is often associated with pancreatic insufficiency at birth. Since it is currently not possible to examine these initiating events of CF pancreatic disease in human, we chose to utilize the sheep model of CF (*CFTR*^*−/−*^) (Fan et al. 2018) that closely recapitulates the in utero phenotypes of human CF pancreatic disease (Van Wettere et al. 2022; Harris 2021), and has many physiological and developmental similarities (reviewed in Banstola and Reynolds (2022); Abi-Nader et al. 2012; Alcorn et al. 1981; Barry and Anthony 2008)). One of the earliest features of human CF in utero, which is evident at about 12 weeks of gestation, is the appearance of mucoid (periodic acid Schiff-positive) material in the pancreatic ducts (Boue et al. 1986). In earlier work we showed that this material contained the MUC6 mucin (Reid et al. 1997) and that *MUC6* gene expression colocalized with that of *CFTR* in pancreatic duct cells (Hyde et al. 1997; Marino et al. 1991). Similarly, in the *CFTR*^*−/−*^ sheep model (Fan et al. 2018; Van Wettere et al. 2022) pancreatic disease with acinar and ductal dilation, mucus obstruction and fibrosis is evident by 80 days of gestation (147 days). There is some controversy about the initiating events in CF pancreatic disease and whether these may involve a developmental defect in duct tubulogenesis (Breton et al. 2016) rather than simple obstruction of the ducts by inspissated secretions. To address the underlying mechanisms, we took a genome-wide approach to identify transcriptional differences between developing WT and *CFTR*^*−/−*^ sheep pancreas at five timepoints through gestation and at term, as described previously (Van Wettere et al. 2022). We dissected pancreatic tissue from the center of the organ (Fig. S1A), extracted RNA and performed bulk RNA-seq. Bioinformatic analysis revealed developmental changes in the pancreas transcriptomic profile across the time points in WT and *CFTR*^*−/−*^ sheep separately. We also identified genes that were differentially expressed between WT and *CFTR*^*−/−*^ sheep pancreas at each timepoint. These data provide a high-quality molecular profile of WT and *CFTR*^*−/−*^ pancreas development and differentiation, and gene ontology (GO) process enrichment analysis using two platforms identified disease-associated changes in multiple biological processes. Next, we performed single-cell RNA-seq (scRNA-seq) on pancreas tissue collected at three gestational time points 80-day, 120-day and term from WT and *CFTR*^*−/−*^ animals to investigate the impact of loss of CFTR function on cellularity and cell identity within the tissue. Accompanying the initiation of aberrant histology in the *CFTR*^*−/−*^ pancreas by 80 days gestation, we observed fewer acini, stromal imbalance, and a cell cluster with marker genes consistent both with activated stellate cells and injured Schwann cells, which was significantly over-represented in the disease state at this time point and at 120 days. At term, the stellate cell population was significantly more abundant in the *CFTR*^*−/−*^ lambs. These scRNA-seq results were confirmed by immunostaining.

Pancreatic stellate cells (PSCs) are multifunctional cells that were first identified in mouse pancreas due to their similarity with the much better defined hepatic stellate cells, and based upon storage of vitamin A/lipid droplets (Watari et al. 1982). Later work isolating and characterizing equivalent star-shaped, lipid-containing cells in rat and human pancreas led to the terminology of PSCs (Apte et al. 1998; Bachem et al. 1998; Ikejiri 1990). PSCs exist in two main states: quiescent (under normal physiological conditions) and activated. Quiescent cells, which play a structural role in the pancreas around acini and vasculature, contain lipid droplets, which stain with Oil-Red O, also desmin and vimentin. In contrast activated cells, which are triggered by many pathological stimuli, lose lipid droplets and acquire a myofibroblast phenotype, expressing alpha smooth muscle actin (αSMA) among other characteristic markers. PSC activation is well studied both in pancreas cancer (Murray et al. 2022; Froeling et al. 2011) and pancreatitis (Yang et al. 2022) (reviewed in Masamune and Shimosegawa (2013); Sherman 2018; Apte et al. 2013)) and though the markers of this process may show some species specificity, the underlying mechanisms are largely conserved. Of note a comprehensive single-cell atlas of the human and mouse pancreas (Baron et al. 2016) identified at least two types of activated stellate cells, the first expressing markers of extracellular matrix more highly, and the second showed high levels of cytokine, interleukin and chemokine gene expression. Our observation of overrepresentation of a unique population of activated stellate cells/injured Schwann cells in the *CFTR*^*−/−*^ sheep pancreas during gestation, together with a significantly greater abundance of stellate cells at term, suggests that loss of CFTR from pancreatic duct cells results in a fibrotic and/or injury response that may be governed by these cells. Hence, CF pancreatic disease mechanisms share key features in common with other pancreatic pathologies including pancreatitis and pancreatic adenocarcinoma. These similarities may indicate novel therapeutic approaches to attenuating or reversing CF pancreatic pathology by targeting these cells.

## Materials and methods

The materials and methods for this study were as described in our previous work (Van Wettere et al. 2022; Kerschner et al. 2023), but are reiterated here for purposes of reproducibility.

*Animals.* American Romney breed of domestic sheep (*Ovis aries*) was used in this study. All animal studies were approved and monitored by the Institutional Animal Care and Use Committee (IACUC) at Utah State University (IACUC protocol # 10089) and conformed to the National Institute of Health guidelines. WT sheep were bred according to standard protocols. Briefly, ewes were synchronized at estrus using an intramuscular (IM) injection of 2.5 ml EstruMate containing 250 μg/ml of cloprostenol, a synthetic analogue of prostaglandin F2α (Merck Animal Health) or Controlled Internal Drug Release (CIDR) (0.3 g prostaglandin) and introduced to the ram on the same day. Ultrasonography around 40 days confirmed pregnancy status and day 1 of gestation was defined as 48 h post EstruMate injection. WT term animals were born naturally and time after birth until euthanasia was recorded. Additionally, heterozygous exon 2 targeted (Fan et al. 2018) (*CFTR*^*+/−*^) ewes were bred to an exon 2 targeted *CFTR*^*+/−*^ ram to produce newborn *CFTR*^*−/−*^ lambs by natural breeding.

### Generation of *CFTR*^*−/−*^ sheep pregnancies by somatic cell nuclear transfer (SCNT)

A. Cloned animals used for bulk RNA-seq experiments were described previously (Kerschner et al. 2023). Briefly, *CFTR*^*−/−*^ sheep were generated by SCNT using genetically modified sheep fetal fibroblasts (SFFs) (CF 60 male, *CFTR*^*−/−*^ exon 2 targeted) derived from American Romney fetuses as described previously (Fan et al. 2018). SCNT recipient ewes were synchronized prior to embryo transfer as described elsewhere (Yang et al. 2016) with the following modification: CIDR devices containing 0.3 g of progesterone were used and 2.0 mL of EstruMate was given IM at the time of CIDR removal. A minimum of two fetuses were recovered at days 50, 65, 80, 100, 120 of gestation and 147–150 day (term) for histological and molecular analysis as described below. All lambs were genotyped immediately after birth using PCR/RFLP assay and Sanger sequencing as previously described (Fan et al. 2018) to confirm the *CFTR*^*−/−*^ animals. The number of fetuses analyzed was limited by the stochastic frequency of singleton, twin and triplet pregnancies, the need to collect all animals for bulk RNA-seq in a single breeding season to minimize natural variation, and the high cost of large animal work. However, since all sheep were inbred American Romney, we expect natural variation to be less than in human tissues.
B. For scRNA-seq experiments: a male neonatal fibroblast cell line (CF2503, exon 2 targeted *CFTR*^*−/−*^) was used for SCNT as described previously (Fan et al. 2018). In total, 290 cloned embryos were transferred into 22 estrus synchronized recipients. Eleven pregnancies were initially established as confirmed by ultrasonography at 40 ± 3 days of gestation (11/22 = 50%). The following samples were collected at various gestational time points: three fetuses collected from two recipients at day 80 of gestation, 5 fetuses from 3 recipients at day 120 of gestation (one fetus had large offspring syndrome and was not used for subsequent analysis) and one pregnancy went to term and was used for newborn sample collection. As previously PCR/RFLP and Sanger sequence results (Fan et al. 2018) indicated that all cloned fetuses/lambs carried the same mutations as those of the donor cells they originated from. The late-term abortion rate was 22.7% (5/22*)*, which is consistent with other sheep cloning data (Davies et al. 2022).

### Histopathologic analysis and immunochemistry

A necropsy was performed on all fetuses collected, to examine for gross lesions and the findings were documented as described previously (Van Wettere et al. 2022). Pancreatic tissue samples were collected and fixed in 10% neutral buffered formalin for histology. Formalin-fixed tissue sections were processed and embedded in paraffin according to routine histologic techniques. Sections, 5-µm thick, were stained with hematoxylin and eosin (H&E), alcian blue, or periodic acid–Schiff (PAS) stain according to standard methods and examined by light microscopy. For immunostaining of sections, tissues were deparaffinized and rehydrated, followed by antigen retrieval with sodium citrate buffer (10 mM sodium citrate and 0.05% Tween 20, pH 6.0) in a 98 °C water bath for 45 min. The sections were then post-fixed in 4% paraformaldehyde (in PBS) for 15 min, permeabilized with 0.5% saponin for 10 min, blocked with 3% BSA, and stained by standard protocols. Briefly, the sections were first incubated with primary antibodies [collagen I (Abcam ab34710), alpha smooth muscle actin (Abcam ab7817), SPARC (Abcam ab290636) and cleaved caspase-3 (Cell Signaling #9661); all at 1/100 dilution in PBS containing 0.1% BSA] overnight at 4 °C. After three washes with PBS containing 0.05% tween 20 (PBS-T), the sections were incubated with secondary antibodies [Alexa Fluor 488 AffiniPure donkey anti-rabbit IgG (H + L) or Rhodamine Red-X (RRX) AffiniPure Donkey Anti-Mouse IgG (H + L), at 1: 400 dilution; Jackson ImmunoResearch] at room temperature for 1 h in the dark. After three further washes with PBS-T, the specimens were counterstained with DAPI and mounted with mounting medium (Vector laboratories H-1700). The terminal deoxynucleotidyl transferase-mediated dUTP nick-end labelling (TUNEL; Invitrogen C10619) assay and cleaved caspase-3 [cleaved caspase-3 (Asp175) antibody staining at 1/100 dilution; Cell Signaling, #9661] immunohistochemistry were performed simultaneously according to the manufacturer’s protocol.

### Tissue collection for RNA processing

Simultaneously with tissue collection for histology, pancreas tissue samples (defined in Fig S1A) were snap frozen in liquid nitrogen and stored at − 80 °C for RNA purification. All tissues collected for bulk RNA-seq of *CFTR*^*−/−*^ animals were from cloned animals. Tissues were homogenized in TRIzol (Thermo Fisher, 15596018) in a dry ice-cooled MP Biomedicals FastPrep-24 Classic homogenizer using Lysing Matrix D tubes (MP Biomedicals, 116913100) for 1 or 2 × 20 s cycles at 4.0 m/s. RNA was isolated using the TRIzol manufacturer's protocol. RNA quality was assessed by NanoDrop, formaldehyde gel electrophoresis and Tapestation prior to choosing high quality samples for RNA-seq.

### RNA sequencing (RNA-seq) and data analysis

RNA samples passing strict QC criteria were used as templates for cDNA library synthesis with oligo dT priming, and amplified libraries were pooled and sequenced (100 bp paired end) on a NovaSeq 6000 machine. Raw sequence reads were aligned to the Oar_v4.0 (oviAri4) genome of the Texel sheep breed with STAR version 2.7.0e_0320 (https://github.com/alexdobin/STAR) (Dobin et al. 2013). Aligned reads were assigned to genomic features with featureCounts version 1.6.3 in the Subread package (http://subread.sourceforge.net/) (Liao et al. 2014). Gene expression analysis to identify differentially expressed genes (DEGs) was performed using edgeR version 3.34.1 (Chen et al. 2016). Genes were filtered by the default minimum count defined by library sizes and then normalized by tagwise dispersion. DEGs were filtered for those with a > 1.5 or < -1.5 fold change (log_2_ ± 0.58) and an adjusted p-value ≤ 0.01. For term animals where a large number of DEGs passed the p-value ≤ 0.01 filter and p-value ≤ 0.001 was also used. Data are deposited at GEO: GSE254376.

### Gene ontology analyses

DEGs were filtered to enrich for genes with a fold change ≥ 1.5 and negative binomial adjusted p-value ≤ 0.01 or ≤ 0.001 as specified. RNA-seq gene lists were read into gProfiler (version: e108_eg55_p17_39cdea3), or DAVID gene ontology program and database (DAVID 2021 v2023q1) for individual WT, *CFTR*^*−/−*^ time courses and WT vs *CFTR*^*−/−*^ at each time point, p ≤ 0.01; (Reimand et al. 2016; Raudvere et al. 2019; Huang et al. 2009; Sherman et al. 2022). RNA-seq gene lists for the same conditions at p ≤ 0.001 were read into gProfiler (version: e109_eg56_p17_1d3191d) and DAVID using the same version as above. gProfiler and DAVID were run with default parameters after selecting ‘*Homo sapiens*’ with Benjamini–Hochberg correction for multiple testing. *Ovis aries* DEG lists were run against *Homo sapiens* GO terms since the updated Oar_v4.0 genome is not currently incorporated into the database (June 2023) leading to fewer biological process results.

### Single-cell RNA sequencing (scRNA-seq)

#### Isolation of single cells from tissues

Tissues for scRNA-seq were from WT animals at 80-, 120- and 147 days (term) gestation, cloned *CFTR*^*−/−*^ animals at 80-, 120-days and term, and naturally bred *CFTR*^*−/−*^ lambs at 120 -days and term. For single cell isolation from fresh pancreas: ~ 300 mg of tissue was cut into 2–3 mm pieces and subjected to two rounds of digestion. First in 5 ml of collagenase solution (200 U/ml collagenase, 40 IU/ml DNAse I, 250 μg/ml soybean trypsin inhibitor (SBTI), (all from Worthington), 3 mM CaCl_2_), at 37 °C, with continuous stirring for 30–40 min. Alternatively, Roche Liberase TL (Millipore Sigma) containing 100 µg/ml collagenase was used. Tissue clumps were then disassociated by pipetting 10 times in a 5 ml serological pipette and left to settle for 3–4 min at which point the supernatant was collected and stored on ice as cell suspension 1. Second, 5 ml of fresh collagenase digestion solution as above was added to the settled tissue clumps remaining after the first digestion and again stirred at 37 °C for 30–40 min, at which point very little tissue debris remained. The supernatant (cell suspension 2) was pooled with cell suspension 1 and together passed through a cell strainer (200 µm, pluriSelect) to remove any tissue debris. The strained cell suspension was then centrifuged at 300×*g* for 5 min, washed once in HBSS (Sigma Aldrich) + 2% FBS and centrifuged again. 4 ml of 1 × Red Blood Cell Lysis Solution (Miltenyi Biotec 130-094-183) was added to the cell pellet which was vortexed for 5 s and then incubated for 5 min at room temperature. After 10 ml of HBSS with 2% FBS was added, the mixture was centrifuged at 300×*g* for 5 min and the supernatant was removed. The cell pellet was then digested with 3 ml of Accutase (Innovative Cell Technologies, #AT104) for 15–25 min with frequent gentle pipetting with a wide-bore pipette after which time cells were visualized under a microscope. If any cell clumps persisted these were collected by centrifugation and further digested with 3 ml of Accutase for an additional 10–15 min. Accutase was removed by washing the cells with 5 ml HBSS + 2% FBS, followed by centrifugation to collect the cells. The supernatant was discarded, and the cell pellet was resuspended in 0.5–1.0 ml HBSS, passed through a 20-µm mini cell strainer (pluriSelect) and cells counted.

### Single-cell RNA-sequencing analysis

A Approximately 3000–5000 cells were used for scRNA-seq using the 10 × Genomics Chromium Single Cell 3’ Reagent Kit v3, or v3.1. After quality control of both cDNA and final libraries using Tapestation, the libraries were sequenced on a NovaSeq 6000 machine. Reads were aligned to the Oar_v4.0/oviAri4 (Texel) genome using Cell Ranger 3.1.0. Cell-UMI matrices were exported into Seurat v4.0.3 (Hao et al. 2021) and filtered with the default parameters and a seed specified at 88, min.cells = 3, min.features = 200. Each dataset was then filtered with the following parameters: nFeature_RNA > 200, nFeature_RNA < 6000, percent.mt < 25. Ribosomal protein genes were also filtered out. Batch effect correction was then performed using 30 dimensions and 2000 anchor features. Cell neighbors were found using 10 dimensions and unsupervised clustering at a resolution of 0.06 for all tissues. 10 PCA dimensions were reduced using Uniform Manifold Approximation and Projection (UMAP). Seurat was also used to perform differential gene expression analysis using the receiver operating characteristic (ROC) method. Sequence data are deposited at GEO: GSE254376.

Pancreas samples were as follows with post-processing cell number in parentheses and are summarized in Table 1.

**Table 1:** Samples for scRNA-seq analysis

scRNA-seq ID/ (cell #)	Animal #	Genotype	Age	Cloned/natural
AXH033 (3171)	SF1501-1	WT	80d	Natural
AXH066 (1661)	SFWT194-1	*CFTR*^*+/−*^	80d	Natural
AXH067 (1885)	SFWT194-2	WT	80d	Natural
AXH068 (3694)	SFWT194-3	*CFTR*^*+/−*^	80d	Natural
AXH059 (2464)	SFY1722-1	*CFTR*^*−/−*^	80d	Cloned
AXH115 (3571)	SFP2050-1	*CFTR*^*−/−*^	80d	Cloned
AXH116 (3990)	SFP2050-2	*CFTR*^*−/−*^	80d	Cloned
AXH039 (3407)	SF1301-1	WT	120d	Natural
AXH040 (2589)	SF1301-2	WT	120d	Natural
AXH073 (6027)	SFWT1601-2	WT	120d	Natural
AXH079 (5637)	SFY1705A^a^	*CFTR*^*−/−*^	120d	Cloned
AXH080 (4101)	SFY1705B^a^	*CFTR*^*−/−*^	120d	Cloned
AXH121 (2812)	SFY1905-1	*CFTR*^*−/−*^	120d	Cloned
AXH122 (1612)	SFY1905-2	*CFTR*^*−/−*^	120d	Cloned
AXH127 (3629)	SFP2077	*CFTR*^*−/−*^	120d	Cloned
AXH130 (5186)	SFCF2502-1	*CFTR*^*−/−*^	120d	Natural
AXH131 (4462)	SFCF2502-2	*CFTR*^*−/−*^	120d	Natural
AXH036 (2090)	SF173-1	WT	Term	Natural
AXH092 (11676)	SFWT2011-1	WT	Term	Natural
AXH136 (4009)	SFWT2281	WT	Term	Natural
AXH139 (4176)	SFWT1615	WT	Term	Natural
AXH087 (5993)	CF2103A^a^	*CFTR*^*−/−*^	Term	Natural
AXH088 (6490)	CF2103B^a^	*CFTR*^*−/−*^	Term	Natural
AXH112 (6403)	SFCF2114	*CFTR*^*−/−*^	Term	Natural
AXH142 (3452)	SFP2058A^a^	*CFTR*^*−/−*^	Term	Cloned
AXH143 (3502)	SFP25058B^a^	*CFTR*^*−/−*^	Term	Cloned

^a^Denotes 2 separate samples from one animal

B. *Pseudotime analysis* was performed in R (version 4.4.0) using the Monocle3 package (version 1.4.17) (https://cole-trapnell-lab.github.io/monocle3). Eleven  wild type (WT) (4 at 80-days, 3 at 120-days of gestation and 4 at term) and thirteen *CFTR*^*−/−*^ (3 at 80-days, 5 at 120-days of gestation and 5 at term) samples were separately run through the same pipeline.
  For each of the samples, contents of the *filtered_gene_bc_matrices* directory of the *cellranger count* command were used as an input and converted to a Monocle3 object using the *load_mm_data* command. Then samples for each of the runs (WT or *CFTR*^*−/−*^) were combined, the resulting object normalized by log and size factor, and the lower dimensional space calculated using the PCA method. The number of principal components (PC) to use in downstream analysis was found using an elbow plot, resulting in 25 PC used for both WT and *CFTR*^*−/−*^ samples.

The samples were consequently “aligned” to compensate for batch effects using *align_cds* command and UMAP reduced dimensions calculated. Clustering was then performed using the *cluster_cells* command with a resolution of 1e-6 and 10 nearest neighbors for WT and 1e−5 and 20 nearest neighbors for *CFTR*^*−/−*^ and “trajectory” of gene expression change (pseudotime trajectory) found using the *learn_graph* command with default arguments, thus learning a disjointed graph in each partition.

To find pseudotime start points, starting principal points (root principal nodes) were manually identified by picking root nodes in each partition with the largest number of earlier gestation time point cells surrounding them. Then, cells were ordered in pseudotime using the found principal nodes using the *order_cells* command.

### Statistics

Statistical methods are built into the single-cell analysis pipelines as described above.

### Study approval

All animal studies were approved and monitored by the Institutional Animal Care and Use Committee (IACUC) at Utah State University (IACUC protocol # 10089) and conformed to the National Institute of Health guidelines.

## Results

### Developmental transcriptome of WT and *CFTR*^*−/−*^ sheep pancreas

We showed a distinct pattern of histological changes in the ovine *CFTR*^*−/−*^ pancreas by 80 days gestation (Van Wettere et al. 2022). At term the pancreas shows acinar atrophy with stromal collapse, loss of acinar tissue, and also dilation of acini and intralobular ducts with mucus (Fig. S1B). To examine the initiating events, RNA-seq was performed on pancreatic tissues (the region as defined in Fig. S1A) from WT and *CFTR*^−/−^ animals (2 of each) at 50, 65, 80, 100, 120-day animals during gestation and 147-day (term). Principal component analysis (PCA) of RNA-seq data is shown in Fig. S2, where samples are largely seen to cluster by developmental time point. The predominant principal component was defined by the progression along the time course. Two outliers on the PCA plot (d120 WT 2 and *CFTR*^*−/−*^ (CF) d100 2) may be accounted for by minor differences in the cellularity of the tissue and/or in the RIN (RNA integrity) value of RNA samples. Not surprisingly given the profound tissue destruction in the *CFTR*^*−/−*^ term pancreas, the RIN values were slightly lower than those of WT pancreas RNA samples, but still met criteria for RNA-seq (~ RIN > 7).

### Differentially expressed genes through gestation in WT sheep pancreas

**Fig. 1:**
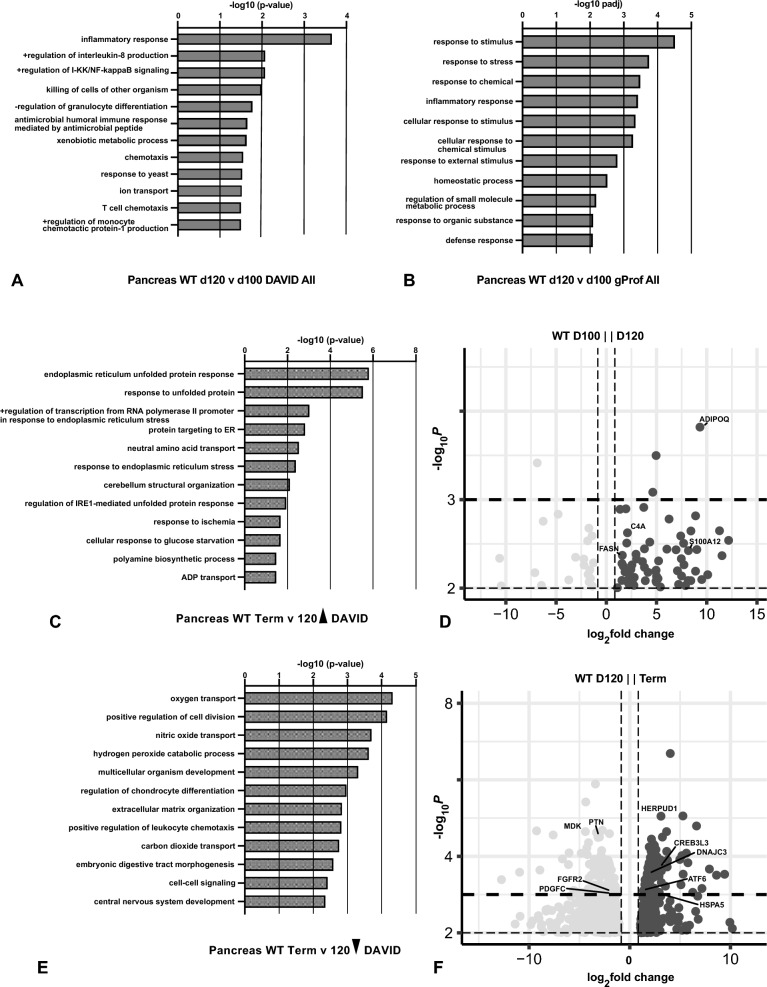
Gene ontology process enrichment analyses of differentially expressed genes between gestational time points in WT sheep pancreas. Differentially expressed genes (DEGs) were filtered to enrich for genes with a fold change ≥  ± 1.5 and Benjamini–Hochberg adjusted p-value ≤ 0.01 for all samples except term where a p-value of ≤ 0.001 was used. Gene ontology analysis by DAVID (**A**, **C**, **D**) or gProfiler (**B**) and up to the top 20 biological processes (BP) are shown. A, B, BP from all DEGs (20 downregulated and 61 upregulated) at 120 days compared to 100 days; **C** BP from genes (399) upregulated at term compared to 120 days; **E** BP from genes (656) downregulated at term compared to 120 days. **D**, **F** Volcano plots (− log_10_ p-value against log_2_ fold change) show DEGs between WT pancreas tissue at (**D**) 100 days and 120 days, and (**F**) 120 days and term. Genes noted in the results are marked

First, to establish the normal course of gene expression through development we conducted pairwise comparisons of differentially expressed genes (DEGs) between each time point of gestation in WT sheep pancreas (Table S1). DEGs were identified using a p-value filter of ≤ 0.01 and log_2_fold change of ± 0.58 (± 1.5 fold change). We compared 65 days to 80 days, 80 days to 100 days, 100 days to 120 days, and 120 days to term. Gene Ontology (GO) process enrichment analysis was then performed using DAVID and g:Profiler on the upregulated and downregulated genes across gestation to determine processes that change with time. For all analyses the sheep DEGs were compared to human GO processes rather than sheep as the latter are not well annotated in databases. Where possible sheep LOCs (assigned to Gene IDs before orthologs of the gene were identified) were manually converted to equivalent human loci, though some were lost in the GO analysis. First, we considered GO processes identified from the list of all DEGs at each time point and then separately upregulated and downregulated DEGs (Table S2A). Between 65 and 80 days gestation there were very few significant DEGs and no associated biological processes called by either DAVID or g:Profiler. Between 80 and 100 days both programs identified biological processes associated with the unfolded protein response, which were driven by few upregulated genes including homocysteine inducible ER protein with ubiquitin like domain 1 (*HERPUD1*), heat shock protein family A (Hsp70) member 6 (*HSPA6*), BAG cochaperone 3 (*BAG3*) and DnaJ heat shock protein family (Hsp40) member B1 (*DNAJB1*) in DAVID, and more genes associated with response to stress/response to stimulus in g:Profiler. Between 100 and 120 days a somewhat larger DEG list identified inflammatory response, positive regulation of interleukin-8 production, and positive regulation of the I-kappaB kinase/NF-kappaB signaling biological processes in DAVID (Fig. 1A). Though marked upregulation of some inflammatory markers such as complement 4A (*C4A*) (see volcano plot in Fig. 1C) was seen in both 120-day animals, others such as fatty acid synthase (*FASN*) and S100 calcium bonding protein A12 (*S100A12*) were particularly strongly upregulated in one animal. Of note pancreatic tissue from the same animal had abundant expression of the adiponectin, C1Q and collagen domain containing (*ADIPOQ*) gene, which is thought to be expressed exclusively in adipocytes, suggesting adipose tissue contamination of this sample. Using the same DEG list, g:Profiler identified 11 biological processes the most significant of which were response to stimulus, response to stress, response to chemical, and inflammatory response (Fig. 1B). Since a large number of DEGs were observed between 120 days and term pancreas with the p-value ≤ 0.01 filter (see volcano plot in Fig. 1F) we increased the stringency for this comparison to a p-value ≤ 0.001 filter (Table S2B). The most significant biological processes identified by DAVID included, endoplasmic reticulum (ER) unfolded protein response (UPR)/response to unfolded protein (Fig. 1C, upregulated genes), oxygen/nitric oxide transport, and positive regulation of cell division (Fig. 1E, downregulated genes). Except for *HERPUD1*, the DEGs driving the UPR processes were different from those seen in the same pathways identified between 80 and 100 days and included *HSPA5,* activating transcription factor 6 (*ATF6*), *DNAJC3* and cAMP responsive element binding protein 3 Like 3 (*CREB3L3*) all of which were greatly upregulated (Fig. 1F). ER stress is known to activate the cleavage of CREB3L3 (previously CREBH), which in turn initiates an acute inflammatory response (Zhang et al. 2006). The DEGs driving the oxygen/nitric oxide transport pathways were hemoglobin subunits, all of which were substantially downregulated at birth, as were the growth factors associated with the positive regulation of cell division process. These included midkine (*MDK*), platelet derived growth factor (*PDGFC*), pleiotropin (*PTN*) and fibroblast growth factor receptor 2 (*FGFR2*). g:Profiler also identified response to UPR as the most significant biological process from the 120 day to term DEG list (upregulated genes, Fig. S3A), but also multiple processes of cell communication and morphogenesis (downregulated genes Fig. S3B). Thirty-five DEGs were associated with the anatomical structure morphogenesis biological process (Table S2B). It is notable that we did not detect biological processes of acinar development or acinar cell differentiation in the developmental time course of WT lambs. This may be due to these processes being well established by 65 days gestation, as evidenced by high levels pancreatic enzyme gene transcripts.

### Differentially expressed genes through gestation in* CFTR*^*−/−*^sheep pancreas

**Fig. 2:**
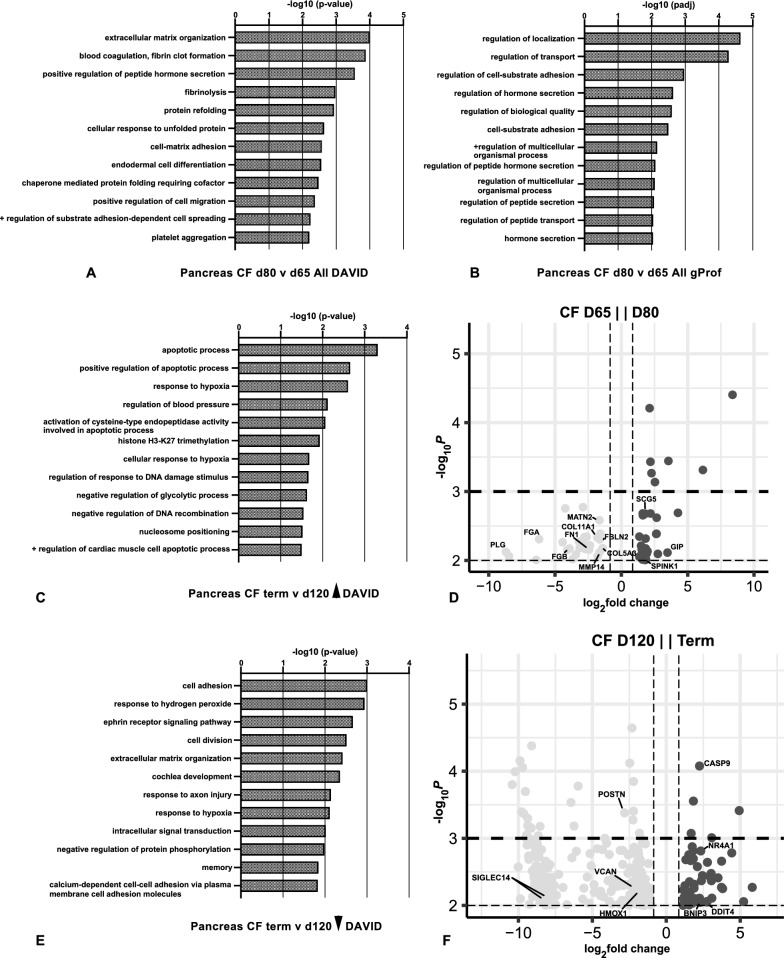
Gene ontology process enrichment analyses of differentially expressed genes between gestational time points in *CFTR*^−/−^ sheep pancreas. Differentially expressed genes (DEGs) were filtered to enrich for genes with a fold change ≥  ± 1.5 and Benjamini–Hochberg adjusted p-value ≤ 0.01 for all samples. Gene ontology analysis by DAVID (**A**, **C**, **E**) or gProfiler (**B**) and the top 20 biological processes (BP) are shown. **A**, **B** BP from all DEGs at 80 days compared to 65 days (27 downregulated and 31 upregulated); **C** BP from genes (49) upregulated at term compared to 120 days; **E** BP from genes (246) downregulated at term compared to 120 days. **D**, **F** Volcano plots (− log_10_ p-value against log_2_ fold change) show DEGs between *CFTR*^*−/−*^ pancreas tissue at (**D**) 65 days and 80 days, and (**F**) 120 days and term. Genes noted in the results are marked

Compared to WT pancreas tissue, many more DEGs were identified between early gestational time points in the *CFTR*^*−/−*^ sheep (Table S3). Since the pancreas is very small and not always clearly identified at 50 days gestation, we started our transcriptomic analysis at 65 days as for WT animals. First performing gene ontology analysis on DEGs between 65 and 80 days using a p-value ≤ 0.01 filter identified many biological processes although many of them were driven by a small number of genes (Table S4, Fig. 2A, B). Extracellular matrix organization was a biological process associated with down regulation of collagens (*COL11A1* and *COL5A3*), fibulin 2 (*FBLN2*), matrilin (*MATN2*), and matrix metalloprotease 14 (*MMP14*) genes among others between 65 and 80 days (see volcano plot in Fig. 2D). Significant down regulation of fibronectin (*FN1*), fibrinogen beta (*FGB*) and alpha (*FGA*) chains and plasminogen (*PLG*) between 65 and 80 days caused the biological processes of blood coagulation to be identified by DAVID (Fig. 2A). However, although *FN1* showed a reduction in both animals the *FGB*, *FGA* and *PLG* levels represent extremely high levels of these clotting factors in only one animal at 65 days. Biological processes identified by both GO platforms also included regulation of hormone/peptide hormone secretion which in g:Profiler (Fig. 2B) were associated with up regulation of secretogranin 5 (*SCG5*), cellular communication network factor 3 (*CCN3/NOV*), free fatty acid receptor 3 (*FFA3R*), serine peptidase inhibitor Kazal type 1 (*SPINK1*) and gastric inhibitory peptide (*GIP*) (Fig. 2D). Of particular interest is the upregulation of *SPINK1,* which encodes a trypsin inhibitor protein that is secreted from pancreatic acinar cells and prevents premature activation of pancreatic zymogen granules by trypsin (Greene et al. 1976). Also of note, GIP stimulates insulin secretion (Dupre et al. 1973). Between 80- and 100-days gestation the biological processes identified are largely based on a small number of DEGs and are of rather low significance. The most significant in the DAVID analysis (Fig. S4A) are response to hypoxia, negative regulation of transcription from RNA polymerase II promoter, and positive regulation of cell migration. In g:Profiler only 3 processes are called including regulation of biological processes/biological regulation and regulation of response to stimulus, though again the statistical significance is low. Next comparing day 120 to day 100 *CFTR*^*−/−*^ pancreas, although twenty biological processes were identified by DAVID from the list of DEGs, including B cell differentiation and adaptive immune response, most of these included only a few genes and the DEG list was biased by one 100-day animal which had particularly high expression of multiple genes in these pathways (Fig. S4B). None of these processes were identified by g:Profiler. In addition to the immune response processes, down regulated genes identified potassium ion transport as a significant process in DAVID, and this was also an artifact of high gene expression in the same 100-day animal. The relatively large number of biological processes (~ 50) identified from DEGs between 120 days and term when using the same p-value ≤ 0.01 filter that we employed at earlier time points encouraged us to again use a more stringent filter of p-value ≤ 0.001. However, unlike in WT pancreas across this development time period very few biological processes reached significance in the gene ontology analysis (data not shown) and were driven by a small number of genes. Considering the processes called from DEGs at p-value ≤ 0.01 (Fig. 2C, D), and then separating those associated with upregulated and downregulated genes, among the most significant in DAVID are apoptosis, response to hypoxia and regulation of blood pressure (upregulated genes, Fig. 2C), and cell adhesion/extracellular matrix (ECM) organization, response to hydrogen peroxide, ephrin receptor signaling and cell division (downregulated genes, Fig. 2E). Significant downregulated genes driving the ECM organization process include versican (*VCAN*), periostin (*POSTN*) and sialic acid binding Ig like lectin 14 (*SIGLEC14*) while heme oxygenase 1 (*HMOX1*) contributes to the response to hydrogen peroxide process (see volcano plot in Fig. 2F). Of note, a repression of cell division between 120 days and term was also seen during our previous analysis of sheep lung development (Kerschner et al. 2023). The increase in expression of genes associated with apoptosis, such as caspase 9 (*CASP9*) and nuclear receptor subfamily 4 group A member 1 (*NR4A1*) was evident in the pancreas of both term *CFTR*^−/−^ animals (Fig. 2F), though other genes including BCL2 interacting protein 3 (*BNIP3*) and DNA damage inducible transcript 4 (*DDIT4*) were dramatically upregulated in only one animal.

### Differentially expressed genes comparing WT and *CFTR*^*−/−*^ animals at specific timepoints through gestation

**Fig. 3:**
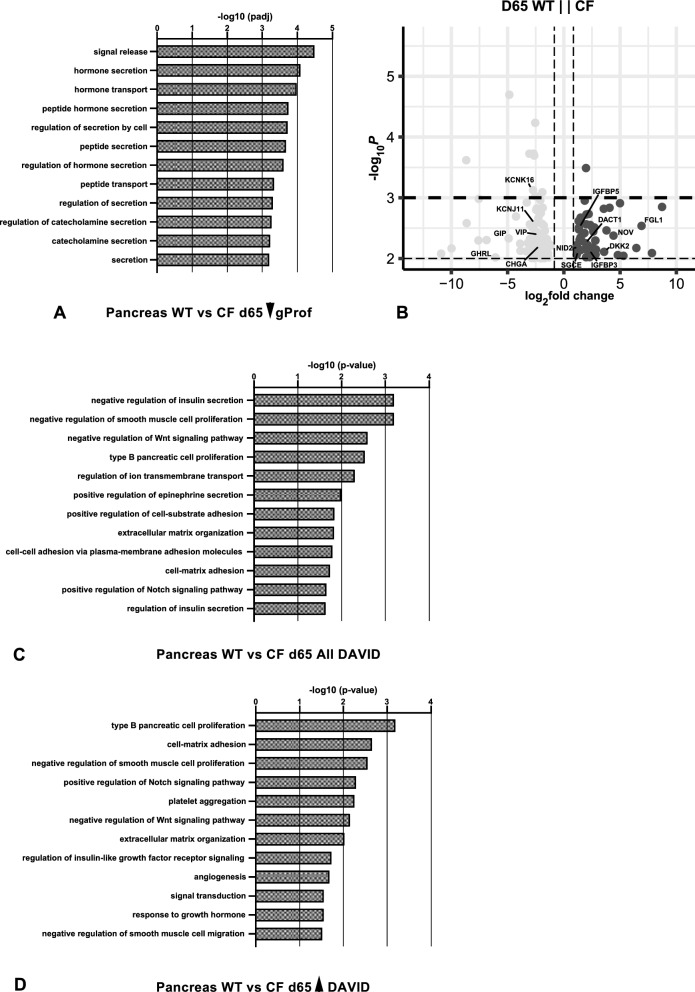
Gene ontology process enrichment analyses of differentially expressed genes between WT and *CFTR*^−/−^ sheep pancreas at individual gestational time points. Differentially expressed genes (DEGs) were filtered to enrich for genes with a fold change ≥  ± 1.5 and Benjamini–Hochberg adjusted p-value ≤ 0.01 for all samples. Gene Ontology analysis by gProfiler (**A**) or DAVID (**C**, **D**) and up to the top 20 biological processes (BP) are shown. **A** BP from genes downregulated at 65 days in *CFTR*^−/−^ compared to WT; **C** BP from all DEGs comparing WT and *CFTR*^−/−^ at 65 days; **D** BP from genes upregulated at 65 days in *CFTR*^−/−^ compared to WT. **B** Volcano plot (-log_10_ p-value against log_2_ fold change) shows DEGs between *CFTR*^−/−^ compared to WT at 65 days with a p-value ≤ 0.01 (80 genes downregulated and 52 upregulated)

Next, we compared DEGs between WT and *CFTR*^−/−^ pancreas through gestation (Table S5). At 65 days a dramatic difference was observed between the two genotypes, with down-regulated genes in the *CFTR*^−/−^ pancreas identifying similar biological processes in both DAVID and g:Profiler (Fig. 3A, 3C, Fig. S5A) including hormone secretion, regulation of insulin secretion and peptide transport/peptide hormone processing (Fig. 3A, 3B). Much lower abundance transcripts of ghrelin and obsestatin prepropeptide (*GHRL*), gastric inhibitory polypeptide (*GIP*), vasoactive intestinal peptide (*VIP*), chromogranin A (*CHGA*) and potassium inwardly rectifying channel subfamily J member 11(*KCNJ11*) in the 65 day.

*CFTR*^−/−^ pancreas compared to WT were among genes contributing to these processes (see volcano plot in Fig. 3B). Ghrelin regulates gastric acid secretion, gastrointestinal motility and pancreatic glucose-stimulated insulin secretion, among other functions (Napolitano et al. 2018). *GIP* and *VIP* encode glucagon superfamily proteins (Gremlich et al. 1995; Chandra and Liddle 2014) the former has a key role in glucose homeostasis through stimulation of insulin secretion from pancreatic beta cells and the latter increases glycogenolysis and relaxes stomach and gall bladder smooth muscle. Chromogranin A is a precursor of pancreastatin (and other active peptides), which is found in secretory vesicles in the endocrine pancreas where it inhibits glucose-induced insulin release (Wollam et al. 2017). *KCNJ11* encodes a potassium channel that is necessary for normal regulation of insulin secretion (Fournet and Junien 2003). Also greatly downregulated in the *CFTR*^−/−^ pancreas compared to WT is potassium two pore domain channel subfamily K member 16 (*KCNK16*) which encodes an outwardly rectifying potassium channel notably involved in the pancreas where it is activated at alkaline pH (Han et al. 2003). In contrast, insulin like growth factor binding protein 3 and 5 (*IGFBP3*, *IGFBP5*) and cellular communication network factor 3 (*CCN3*/*NOV*) which are all significantly upregulated in *CFTR*^−/−^ pancreas compared to WT (Fig. 3B) are associated in the DAVID analysis with biological processes of type B pancreatic cell proliferation and negative regulation of smooth muscle cell proliferation (Fig. 3C). Of note, these genes are also identified in g:Profiler associated with biological processes of regulation of cell migration and motility (Fig. S5B). Other upregulated DEGs include dickkopf WNT signaling pathway inhibitor 2 (*DKK2*) and dishevelled binding antagonist of beta catenin 1(*DACT1*), which are involved in the positive regulation of Notch signaling pathway (Li et al. 2015), and several genes involved in cell–matrix adhesion including sarcogylan epsilon (*SGCE*), fibrinogen like 1 (*FGL1*) and nidogen 2 (NID2) (Fig. 3B, D). Significant variations in extracellular matrix binding and cell adhesion processes are also seen in the g:Profiler analysis (Fig. S5B). Together these DEGs and associated biological processes suggest that at 65 days the pancreas of *CFTR*^−/−^ animals is either immature or damaged compared to WT animals.

The comparison of WT and *CFTR*^−/−^ pancreas at 80 days gestation was not informative as there were very few DEGs and several of these encoded long non-coding RNAs (lncRNAs) of unknown function. At 100 days of gestation, although many biological processes were associated with DEGs including cellular response to interferon gamma, fatty acid transport, cholesterol homeostasis, extracellular matrix organization and negative regulation of transcription from RNA polymerase II promoters, substantial variation in transcript abundance between the two WT pancreas samples and between the two *CFTR*^−/−^ animals biased the results and prevented robust conclusions. Similarly at 120 days gestation substantial variation of the transcriptomes of the two WT pancreas samples was evident and few significant biological processes were identified from the DEG list in DAVID (and none in g:Profiler). However, of considerable interest was the dramatic upregulation of regenerating family member 1 alpha (*REG1A*) in the two *CFTR*^−/−^ animals compared to WT (Table S5). *REG1A* encodes a protein that is secreted by the pancreas and associated with islet cell regeneration, diabetogenesis and pancreatic stone formation in pancreatitis (Hirano et al. 2013; Watanabe et al. 1990). Also substantially upregulated in *CFTR*^−/−^ animals at 120 days (and at 100 days and term) was the FAU ubiquitin-like and ribosomal protein S30 fusion gene (*FAU*) (Table S5), which may be a marker of apoptosis (Pickard et al. 2011).

**Fig. 4:**
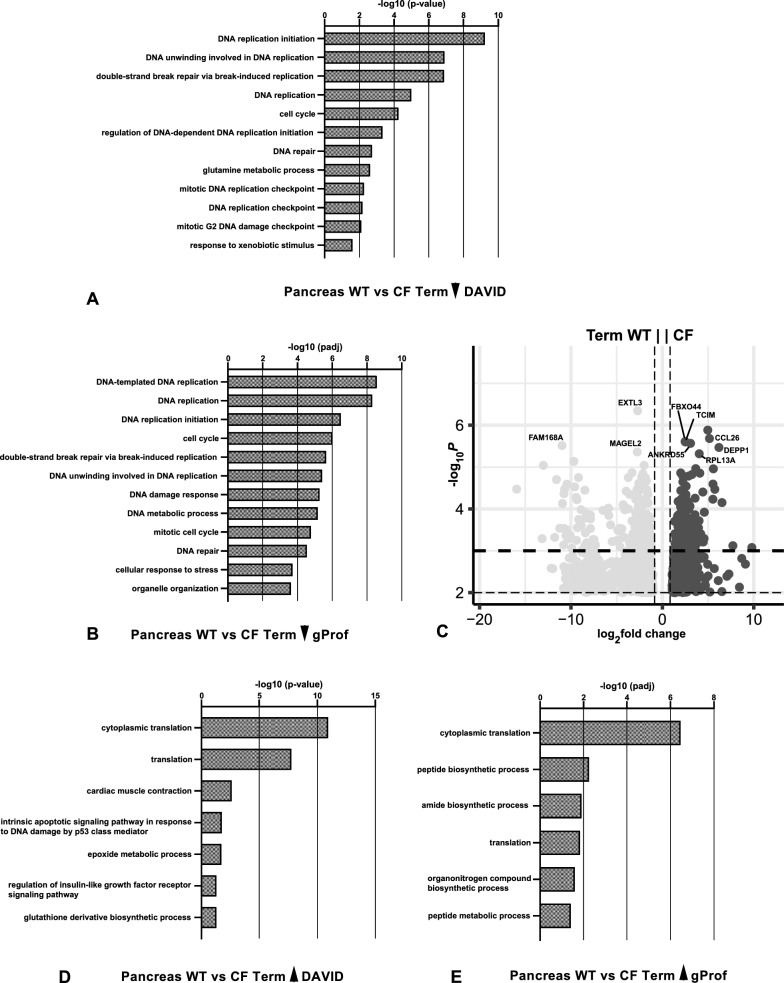
Gene ontology process enrichment analyses of differentially expressed genes between WT and *CFTR*^−/−^ sheep pancreas at individual gestational time points. Differentially expressed genes (DEGs) were filtered to enrich for genes with a fold change ≥  ± 1.5 and Benjamini–Hochberg adjusted p-value ≤ 0.001 for all samples. Gene ontology analysis by DAVID (**A**, **D**) or gProfiler (**B**, **E**) and up to the top 20 biological processes (BP) are shown. **A**, **B** BP from genes (246) downregulated in *CFTR*^−/−^ compared to WT at term; **D**, **E** BP from genes (183) upregulated in *CFTR*^−/−^ compared to WT at term. **C** Volcano plot (− log_10_ p-value against log_2_ fold change) shows DEGs between *CFTR*^−/−^ compared to WT at term with a p-value ≤ 0.01 (936 genes downregulated and 717 upregulated)

Lastly comparing pancreas from WT and *CFTR*^−/−^ animals at term, the gene ontology process enrichment analysis was carried out with p-value ≤ 0.01 filter (Table S6A) (see volcano plot in Fig. 4C) and p-value ≤ 0.001 filters (Table S6B) as so many genes passed the lower threshold. With the p-value ≤ 0.01 and ≤ 0.001 cut offs, DEGs that were downregulated in *CFTR*^−/−^ term pancreas identified biological processes involved in DNA replication and cell division, with highly significant p-values in both DAVID and gProfiler (Fig. 4A, B). This observation is consistent with those in the comparison between *CFTR*^−/−^ and WT sheep lungs at term (Kerschner et al. 2023) and suggests a generalized deficit in these processes in the *CFTR*^−/^ animals at birth. In contrast, genes that were upregulated in *CFTR*^−/−^ animals identified biological processes associated with many components of translation including ribosome assembly in both DAVID and g:Profiler (Fig. 4D, E).

### Altered cell type abundance through gestation in *CFTR*^*−/−*^ fetuses as shown by bulk RNA-seq

**Fig. 5:**
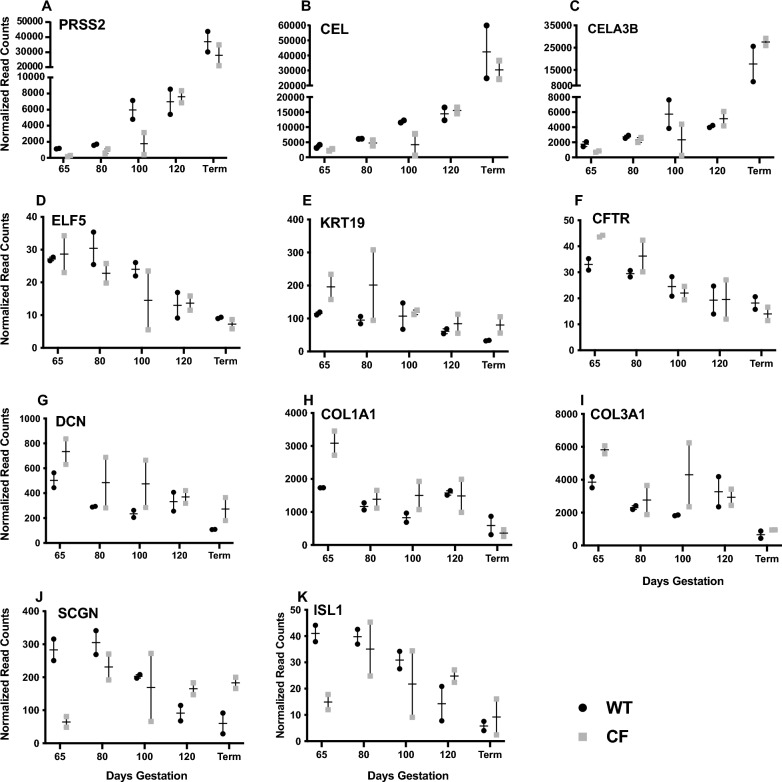
Developmental profile of marker gene expression for pancreatic acinar cells, pancreatic duct cells, stromal cells and endocrine cells in WT and *CFTR*^−/−^ sheep pancreas. Normalized read counts from bulk RNA-seq data show gene expression through gestation at 65-, 80-, 100-, 120-days gestation and at term in WT and *CFTR*^−/−^ sheep. **A**–**C** acinar cell markers *PRSS2*, *CEL* and *CELA3B;* (**D**–**F**) pancreatic duct epithelial cell markers *ELF5*, *KRT19* and *CFTR;* (**G**–**I**) markers of stromal cells *DCN*, *COL1A1*, *COL3A1*; (**J**, **K**) markers of endocrine cells *SCGN* and *ISL1*. WT values are shown has black circles and *CFTR*^*−/−*^ values as grey squares

We next took a gene-centric approach to examine changes in transcript abundance with gestational age for known markers of each major cell type in the developing pancreas. For this analysis we plotted normalized read counts for each gene in the bulk RNA-seq data against days of gestation for the 2 WT and 2 *CFTR*^−/−^ (CF) animals, with standard error of the mean (SEM) shown on the graphs for each sample pair (Fig. 5). The small number of animals precludes formal statistical analysis, so we simply document consistent trends. For acinar markers we chose serine protease 2 (*PRSS2*), chymotrypsin like elastase 3B (*CELA3B*) and carboxyl ester lipase (*CEL*), all of which increased gradually through gestation in both WT and *CFTR*^−/−^ animals with the most marked change between 120 days and term (Fig. 5A–C). These results likely reflect the growth and development of functional acini through gestation. Read counts in the *CFTR*^−/−^ animals were generally slightly lower than WT until 120 days, suggesting early loss of acinar tissue or possibly a slightly delayed acinar development (Fig. 5A–C). For pancreatic duct epithelial cells, we examined E47-like ETS transcription factor 5 (*ELF5*), keratin 19 (*KRT19*) and the cystic fibrosis transmembrane conductance regulator (*CFTR*) gene. Abundance of *ELF5, KRT19* and *CFTR* transcripts slightly decreased through gestation, consistent with a smaller cellular contribution from the ductal tree as the pancreas develops (Fig. 5D–F). The expression of general markers for stromal cells including decorin (*DCN*), collagen type 1 alpha 1 chain (*COL1A1*) and collagen type 3 alpha 1 chain (*COL3A1*), broadly declined through gestation but were somewhat higher in individual *CFTR*^−/−^ animals than WT animals at multiple time points prior to term (Fig. 5G–I). Of note, markers of pancreatic stellate cells including secreted protein acidic and cysteine rich (*SPARC*) and actin alpha 2, smooth muscle (*ACTA2*) were generally higher in *CFTR*^−/−^ animals than WT controls before 80 days gestation though there was substantial variation between individuals (Fig. S6 A, B). Lastly, the transcript abundance of endocrine cell markers secretogranin (*SCGN*) and ISL LIM homeobox 1 (*ISL1*) fall after 80 days through to term but are markedly lower at 65 days in the *CFTR*^−/−^ animals compared to WT, implicating a developmental delay in endocrine differentiation (Fig. 5J-K).

### Comparative single-cell RNA-seq analysis of WT and* CFTR*^*−/−*^sheep pancreas through development

Because bulk RNA-seq can only provide a global readout of gene expression changes in the pancreas and our goal was to determine the cellular origins of pancreatic pathology in the *CFTR*^−/−^ animals, we next performed single-cell RNA-seq (scRNA-seq) on pancreatic tissue from animals at 80 days, 120 days gestation and at term. These data were generated from different animals from those used for bulk RNA-seq analyses above and were collected in subsequent annual sheep breeding cycles, one to three years later.

**Fig. 6:**
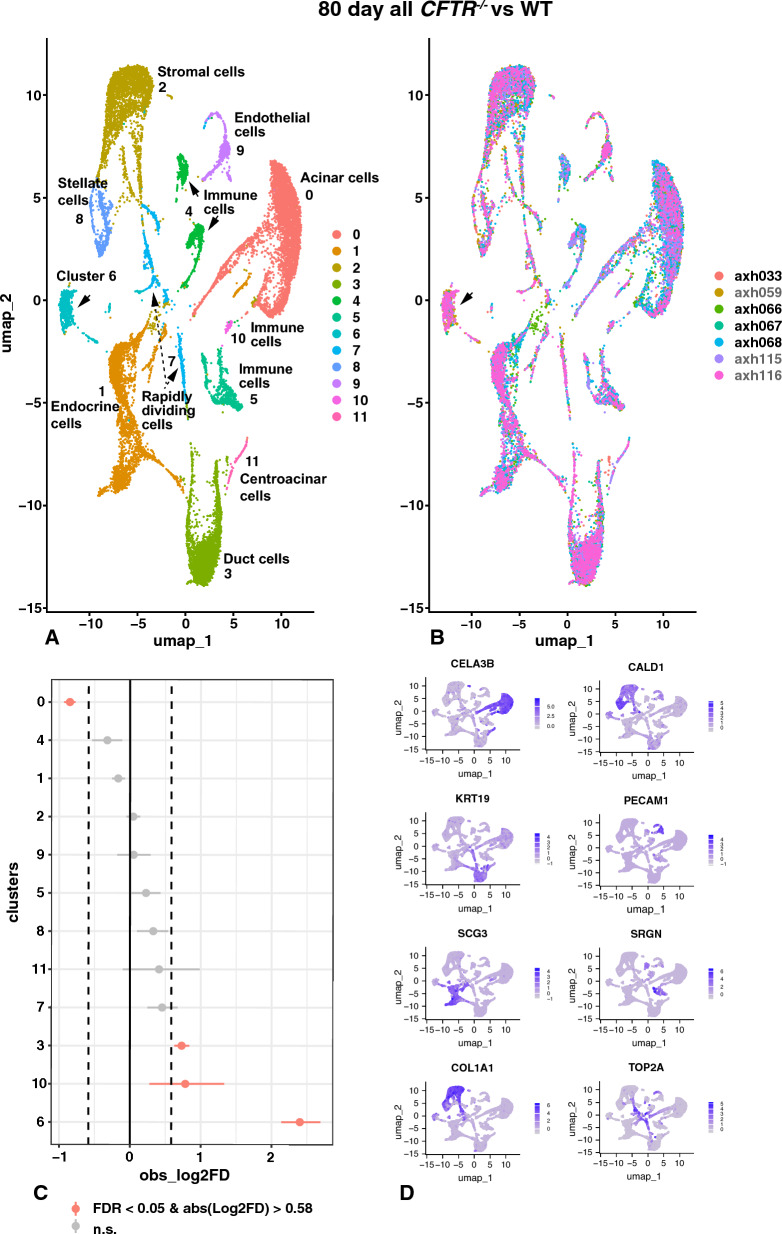
Single-cell RNA-seq defines cell types in 80-day WT and *CFTR*^−/−^ sheep pancreas and identifies an activated stellate cell/injured Schwann cell population that is significantly overrepresented in the *CFTR*^−/−^ pancreas. UMAP plots of merged WT (AXH033, AXH066, AXH067, AXH068) and *CFTR*^−/−^ (AXH059, AXH115, AXH116) sheep pancreas by differential gene expression profiles (**A**) or donor genotype (**B**, black font WT, grey font *CFTR*^−/−^). Twelve cell clusters are seen in (**A**, **B**) including acinar cells, endocrine cells, stromal cells, duct cells, three immune cell clusters, rapidly dividing cells, quiescent stellate cells/myofibroblasts, and activated stellate cells/injured Schwann cells (cluster 6, arrowed). Marker gene lists are shown in Table S7. **C** Single cell proportion test comparing *CFTR*^*−/−*^ with WT shows significant over representation of activated stellate cells/injured Schwann cells and duct cells, and under representation of acinar cells. **D** Feature plots show cluster marker genes for clusters 0–7

At 80 days a total of 10,025 cells from three cloned *CFTR*^*−/−*^ fetuses (AXH059, AXH115 and AXH116) were compared with a total of 10,411 cells from non-CF fetuses (AXH033 and triplets AXH066, AXH067, AXH068) (Table 1). Fetus AXH033 and AXH067 were homozygous WT, while AXH066 and AXH068 were heterozygous for WT and *CFTR*^*−/− (exon11)*^ alleles (Fan et al. 2018). As expected, since CF is an autosomal recessive disease, these heterozygote animals had a completely normal phenotype (confirmed by histology) and were utilized as WTs for comparisons in the scRNA-seq data. The distribution of cell types in the *CFTR*^*−/−*^ and WT tissue samples is shown in Fig. 6 and the associated marker gene list for each cluster is in Table S7. The UMAP plot in Fig. 6A shows identity by cluster and in Fig. 6B identity by donor. Twelve clusters are identified with the most abundant (cluster 0) including acinar cells/glandular secretory cells, endocrine cells (cluster 1), stromal cells (cluster 2), pancreatic duct epithelial cells (cluster 3), immune cells (i) (cluster 4), immune cells ii (cluster 5), a cluster (6) of dual identity, rapidly dividing cells (cluster 7), stellate cells (cluster 8), endothelial cells (cluster 9), immune cells iii (cluster 10) and centroacinar cells (cluster 11). Among the most significant marker genes used to identify each cluster at 80 days gestation in the 7-donor analysis (Fig. 6A) were the following: Cluster 0, acinar cells, carboxypeptidase A1 (*CPA1*), an enzyme that cleaves C-terminal amino acids from ingested proteins, pancreatic lipase related protein 2 (*PNLIPRP2*), which hydrolyses the main plant membrane lipids; chymotrypsin like elastase 1 (*CELA1*) and *CELA3B* (Fig. 6D) which are both zymogen granule components; *CEL*, which encodes an enzyme that hydrolyses cholesterol and lipid soluble vitamin esters; *PRSS2* that encodes anionic trypsinogen, *DNASE1* and aquaporin 8 (AQP8) encoding the major acinar cell water channel (reviewed in Arsenijevic et al. (2019)). Cluster 1 identifies endocrine cells though these are probably immature as different islet cell types do not have separate identities, even when subclustered on their own (data not shown). Highly expressed genes in this cluster include multiple secretogranins (*SCGN*, *SCG3* (Fig. 6D) and *SCG5*) and chromogranin (*CHGA*); transthyretin (*TTR*), which has a key role in vitamin A/retinol transport; and several genes that carry mutations in maturity onset diabetes of the young (MODY) including *KCNK16* (Graff et al. 2021); and the *ISL1* transcription factor (Chen et al. 2013). Of note at this developmental age insulin is not identified in the marker gene list but the insulin transcriptional repressor 1 (*INSM1*) is highly expressed as is glucagon (*GCG*). Cluster 2 are stromal cells/fibroblasts which differentially express a wide range of fibrillar collagen (*COL*) genes including *COL1A1* (Fig. 6D), *COL1A2*, *COL3A1, COL5A2*, *COL6A*1 and *COL6A3*; genes encoding proteins involved collagen assembly such as *DCN* and lumican (*LUM*); *SPARC*, which encodes a protein with a key role in ECM synthesis; and also TIMP metallopeptidase inhibitor 1 and 2 (*TIMP1/2*), which function as inhibitors of matrix metalloproteins involved in ECM degradation. The retinol-binding protein gene (*RBP4)* is a marker for cluster 2 while *ACTA2* is not, suggesting this cluster is not enriched for activated stellate cells. Cluster 3 cells includes pancreatic duct epithelial cells with markers including keratin 8 and 19 (*KRT8*, *KRT19* (Fig. 6D)); matrix metalloprotease 7 (*MMP7*); solute carrier family 4 member 4 (*SLC4A4*), a sodium bicarbonate transporter; transmembrane 4 L six family member 4 (*TM4SF4*) which regulates adhesive and proliferative status of intestinal epithelial cells (Wice and Gordon 1995); the epithelial ETS factors ETS homologous factor *EHF* and *ELF5*; several claudins (*CLDN4* and *CLDN6*); and *CFTR*. Multiple types of duct epithelial cells or immature cells are likely present in this cluster and the marker gene list includes some unexpected genes. Three distinct clusters (4, 5, and 10) of immune cells are seen which share some classical immune markers such as serglycin, *SRGN* (Fig. 6D) but also each have their own identity. However, since the immune system is quite immature at 80 days, precise cluster characterization may not be possible. Markers for cluster 4 are consistent with B-lymphocytes and include immunoglobulin heavy chain mu (*IGHM*), *CD37*, *CD74*, *CD79A* and *CD79B*, major histocompatibility complex, class II, DQ beta 1 (*DQB*) and lim domain containing 2 (*LIMD2*). Indicative of myeloid cells and specifically macrophages, markers for cluster 5 include toll like receptor 2 (*TLR2*) and *TLR4*, Fc epsilon receptor (*FCER1G*), C-X-C Motif Chemokine ligand 8 (*CXCL8*), cytochrome B-245 beta chain (*CYBB*), C-type lectin domain containing 5A (*CLEC5A*), and the scavenger receptor cysteine-rich superfamily member CD163 (*CD163*). Immune cells in cluster 10 are clearly antigen presenting cells and several major histocompatibility complex, class II genes (for example *HLA-DO*) and CD8 subunit beta (*CD8B*) are on the marker gene list. Cluster 10 cells differentially express many of the same marker genes as cluster 5 but are clearly distinct and may be dendritic cells. Cluster 7 is suggestive of rapidly dividing cells, with marker genes including several that are associated with cell proliferation and cell division such as topoisomerase ii alpha (*TOP2A* (Fig. 6D)), TPX2 microtubule nucleation factor (*TPX2*), centromeric proteins (*CENPE, CENPF and CENPW*).

In addition to the major stromal/fibroblast cell cluster 2, clusters 6 and 8 also show many differentially expressed fibroblast markers. Cluster 8 are myofibroblast-like pancreatic stellate cells and are marked by abundant transcripts of smooth muscle genes including tropomyosin 2 (*TPM2*), caldesmon 1 (*CALD1* (Fig. 6D)), *ACTA2*, matrix Gla protein (*MGP*), transgelin (*TAGLN*) and endothelin receptor Type A (*EDNRA*). Cluster 8 may contain both quiescent and activated stellate cells. Cluster 6 also has a transcriptome that is consistent with activated pancreatic stellate cells but additionally expresses key markers of Schwann cells. Among the top marker genes is apolipoprotein D (*APOD*) which was identified as a marker of juxtatumoral stromal cells in infiltrating pancreas carcinoma (Ricci et al. 2005). APOD, though a high-density lipoprotein, has more similarity to the lipocalin family member plasma retinol (vitamin A)-binding protein (*RBP4*) (cluster 2) and has little homology with other apolipoproteins. Consistent with other observations (Hrabak et al. 2021) these activated stellate cells in cluster 6 show down regulation of *ACTA2*, which is a top marker of cluster 8 (PSCs) but not a marker of cluster 6 cells. Among other markers only seen in cluster 6 are leucine zipper protein 2 (*LUZP2*), catenin alpha like 1 (*CTNNAL1*), tenascin C (*TNC*), sodium voltage-gated channel alpha subunit 7 (*SCN7A*), SH3 and PX domains 2A (*SH3PXD2A*). *CTNNAL1* encodes a protein that is thought to facilitate actin filament and cadherin binding activity (Xiang et al. 2008) and is known to be highly expressed in normal pancreas but is down regulated in the ASPC-1 cancer cell line upon sodium butyrate treatment (Zhang et al. 1998). Tenascin C was recently shown to be highly upregulated in systemic sclerosis and to be associated with fibrosis (Bale et al. 2023), while SH3PXD2A (formerly TKS5) is involved in extracellular matrix degradation in both normal podosomes/invadopodia and in cancer cells (Blouw et al. 2008). Of note, cluster 6 also shows certain neuronal markers characteristically seen in activated stellate cells including GDNF family receptor alpha 1 (*GFRA1*), neurexin 1 (*NRXN1*) and peripheral myelin protein 22 (*PMP22*), which is also a marker of stromal cells in cluster 2 and stellate cells in cluster 8. However, it is also possible that cluster 6 cells are Schwann cells rather than activated stellate cells, as proteolipid protein 1 (*PLP1*), nerve growth factor receptor (*NGFR*) and S100 calcium binding protein B (*S100B*) are all on the marker gene list and are known to be expressed in pancreatic Schwann cells (Baron et al. 2016). Of note, genes encoding myelin sheath proteins such as myelin basic protein (*MPB*) and myelin-associated glycoprotein (*MAG*) are not on the marker gene list for cluster 6, while markers of injured Schwann cells including forkhead box D3 (*FOXD3*) and inhibitor of DNA-binding (*ID4*) are present (Baron et al. 2016; Lovatt et al. 2022). Gamma-aminobutyric acid type A receptor subunit gamma1 (*GABRG1*) on the marker gene list for cluster 6 is strongly indicative of neuronal cells, however, few cell express the Schwann cell marker *SOX10* (data not shown). A recent analysis of early human pancreatic development by single-cell and spatial transcriptomics between 12–20 post conception weeks (pcw) identified multiple Schwann cell types (Olaniru et al. 2023). This human gestational time window corresponds to 46–77 days in the sheep, so the later time points are close to the 80 days sheep pancreas scRNAseq presented here. Interestingly, the main marker of Schwann cells used in the human developmental data was crystallin alpha B (*CRYAB*), which is not on the marker gene list for cluster 6 in the developing sheep pancreas, but rather a marker of stellate cells in cluster 8. Hence, cells in cluster 6 have a rather unique transcriptome that is consistent both with activated stellate cells and injured Schwann cells. Cluster 9 are endothelial cells, marked by endomucin (*EMCN*), C-type lectin domain family 3 member B (*CLEC3B*), platelet and endothelial cell adhesion molecule 1 (*PECAM1* (Fig. 6D)), protein tyrosine phosphatase receptor type B (*PTPRB*), plasmalemma vesicle associated protein (*PLVAP*), cadherin 5 (*CDH5*), together with von Willebrand factor (*VWF*). Cluster 11 cells are likely centroacinar cells since they are close to duct epithelial cells in the UMAP space but express both acinar cell marker genes and *CFTR* (Fig. 6D, Fig S7).

Notable features of the UMAP plot in Fig. 6B showing cluster identity by donor are the under-representation of acinar cells (cluster 0) and over-representation of ductal cells (cluster 3) and particularly activated stellate cells/injured Schwann cells (cluster 6) among *CFTR*^*−/−*^ cells (marked by arrow). This altered distribution is confirmed in the single cell proportion test show in Fig. 6C, where comparing *CFTR*^*−/−*^ (n = 3) with WT (n = 4) cluster 6 (activated stellate/injured Schwann cells) are the most significantly overrepresented cluster in the *CFTR*^*−/−*^ animals. Of lower (albeit statistical) significance is the underrepresentation of acinar (0) cells and the overrepresentation of duct (3) cells and one cluster of immune cells (10) in the *CFTR*^*−/−*^ animals. These data are consistent with the bulk cell data suggesting that at 65 days gestation the *CFTR*^*−/−*^ pancreas appeared immature compared to WT with lower expression of markers of differentiated acini and islet cells among others.

**Fig. 7:**
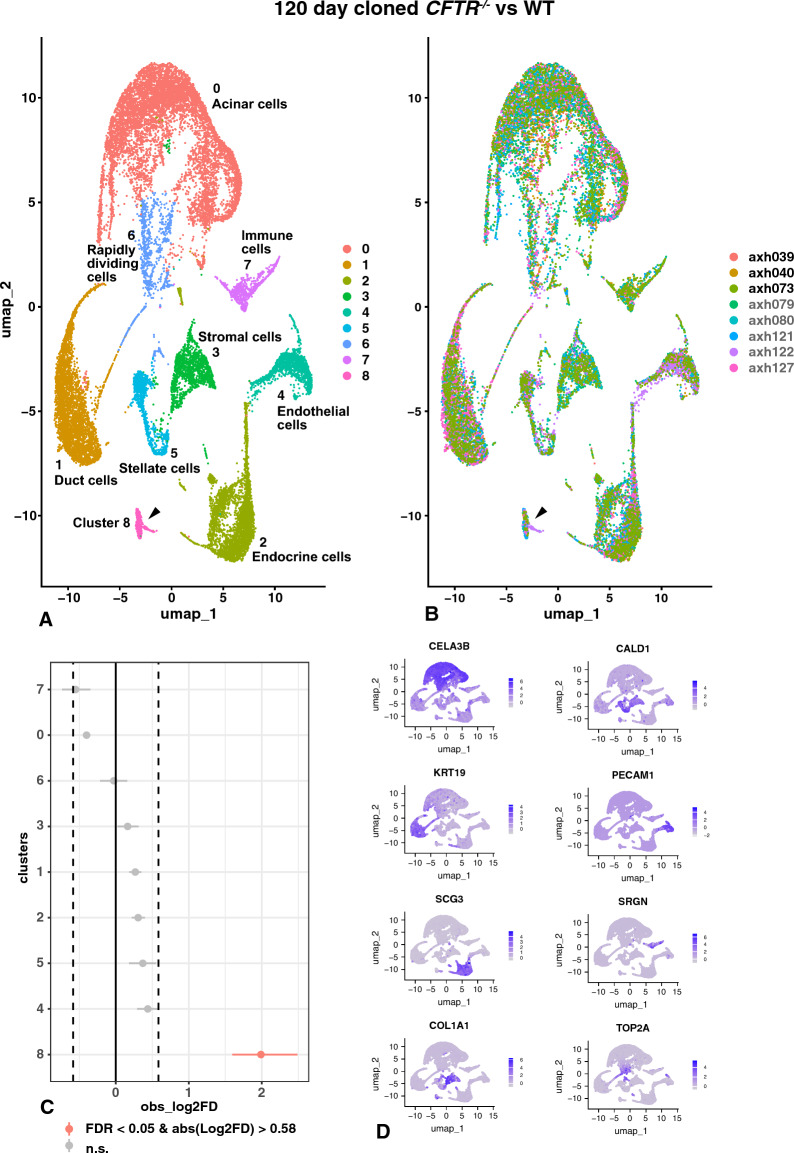
Single-cell RNA-seq defines cell types in 120-day WT and *CFTR*^−/−^ sheep pancreas and identifies an activated stellate cell/injured Schwann cell population that is significantly overrepresented in the *CFTR*^−/−^ pancreas. UMAP plots of merged WT (AXH039, AXH040, AXH073) and *CFTR*^−/−^ (AXH079, AXH080, AXH121, AXH122, AXH127) sheep pancreas by differential gene expression profiles (**A**) or donor genotype (**B**, black font WT, grey font *CFTR*^−/−^). Nine cell clusters are seen in (**A**, **B**) including acinar cells, duct cells, endocrine cells, stromal cells, endothelial cells, immune cells, rapidly diving cells, quiescent stellate cells/myofibroblasts, and activated stellate cells/injured Schwann cells (cluster 8, arrowed). Marker gene lists are shown in Table S8. **C** Single cell proportion test comparing *CFTR*^−/−^ with WT shows significant over representation of activated stellate cells/injured Schwann cells. **D** Feature plots show cluster marker genes for clusters 0–7

Next, we performed scRNA-seq on 120-day gestation pancreas tissue from three WT donor samples (AXH039, AXH040, AXH073) (12, 023 cells) and five samples from *CFTR*^*−/−*^ animals (17,791 cells), two of which were samples processed separately from the same donor (AXH079, AXH080, AXH121, AXH122, AXH127). Cells were also analyzed from two naturally bred 120-day animals (AXH130, AXH131) (9648 cells). Figure 7 shows the 120-day data clustered by identity (Fig. 7A) and by donor (Fig. 7B) and cluster markers are shown in Table S8. As for the 80-day tissue, the most abundant cell type (cluster 0) had marker genes of acinar/exocrine glandular cells, and cluster 0 contained a greater proportion of the total cells than in most animals at 80 days, consistent with the development and differentiation of functional acini during this developmental window. Cluster 0 markers included *PNLIPRP2, CELA3B* (Fig. 7D), *CPA1, PRSS2*, and *CEL,* which were also identified in acinar cells at 80 days, and also phospholipase A2 group 1B (*PLA2G1B*)*,* and *AQP8* among others. Duct epithelial cells are found in cluster 1, and by 120 days the identity is much clearer based upon myAUC values: among the most significant are *CLDN4*, *SLC4A4* and *KRT19* (Fig. 7D), with other markers including *EHF*, *CDLN6*, *CFTR* and *KRT18.* Cluster 2 has markers of endocrine cells, which largely coincide with those seen in endocrine cells at 80-days including genes encoding multiple granins (Herold et al. 2021) *SCG3* (Fig. 7D), *SCG5*, *SCGN* and *SCG2*, *TTR*, *ISL1* and *GCG*. Cluster 3 is one of three that have fibroblast/stromal cell identity and most closely resembles cell cluster 2 at 80 days, with very high expression of multiple collagens including *COL1A1* (Fig. 7D), *COL1A2*, *COL3A1* and *COL6A1* in addition to *DCN*, *LUM* and *SPARC*. *RBP4* is in the marker gene list for cluster 3 cells, but *ACTA2* is not. Endothelial markers identify cluster 4, with high expression of *PECAM1* (Fig. 7D), endothelial cell surface expressed chemotaxis and apoptosis regulator (*ECSCR*), *CDH5*, *PLVAP*, *PTPRB* together with *VWF*, *CD36* and *CD34*. Cluster 5 is another group of fibroblasts, specifically pancreatic stellate cells/myofibroblasts as evidenced by the top marker genes *CALD1* (Fig. 7D) and *TPM*2, which interacts with caldesmon in smooth muscle cells, also *ACTA2*, matrix gla protein (MGP), osteoglycan (OGN), *COL4A1*, *VIM*, *SPARC* and lactate dehydrogenase (LHDA). Cluster 6 appears to be a group of rapidly dividing cells as has been observed in scRNA-seq data from some other human tissues including the lung (Paranjapye et al. 2022). Marker genes for this cluster include topoisomerase ii alpha (*TOP2A* (Fig. 7D)), TPX2 microtubule nucleation factor (*TPX2*), centromeric proteins (*CENPA* and *CENPF*), cell division cycle associated 3 (*CDCA3*) and cyclins (*CCNA2, CCNB1, CCNB2*). The identity of cluster 7 is consistent with immune cells and is most similar to cluster 5 at 80 days. Marker genes of macrophages/mast cells and lymphocytes are seen including *SRGN* (Fig. 7D), *FCER1G*, interleukin receptor subunit gamma (*IL2RG*), pleckstrin (*PLEK*), nuclear factor kappa B subunit 1 (*NFKB1A*) and the REL proto-oncogene, NF-KB subunit (*REL*). Another type of fibroblast is found in cluster 8 that has markers of activated stellate cells, with *NRXN1* and *PMP22* close to the top the list. Additional markers include *COL3A1, COL4A1*, *COL5A3*, and *COL1A2*, *VIM*, the zinc finger E-box homeobox 2 (*ZEB2*) gene, which is a transcriptional repressor, and *SH3PXD2A*. However, as for cluster 6 in the 80-day UMAP plot, the marker genes for cluster 8 also include *APOD*, *PLP1* and *ID4*, but not *MBP* or *MAG* myelin genes. In contrast to cluster 6 cells at 80 days, *CRYAB* is a marker gene for cluster 8 as are myelin protein zero (*MPZ*) and EGF like domain multiple 8 (*EGFL8*), hence these cells may also be injured Schwann cells. Of particular interest is the observation that cluster 8 is largely contributed to by cells from the *CFTR*^*−/−*^ pancreas samples (Fig. 7B and Table S8), moreover in the single cell proportion test (Fig. 7C) cluster 8 is the most significantly overrepresented in the *CFTR*^*−/−*^ samples. Hence, we have additional evidence that the early disease process in the *CFTR*^*−/−*^ pancreas is associated with enrichment of a population of activated stellate cells/injured Schwann cells.

**Fig. 8:**
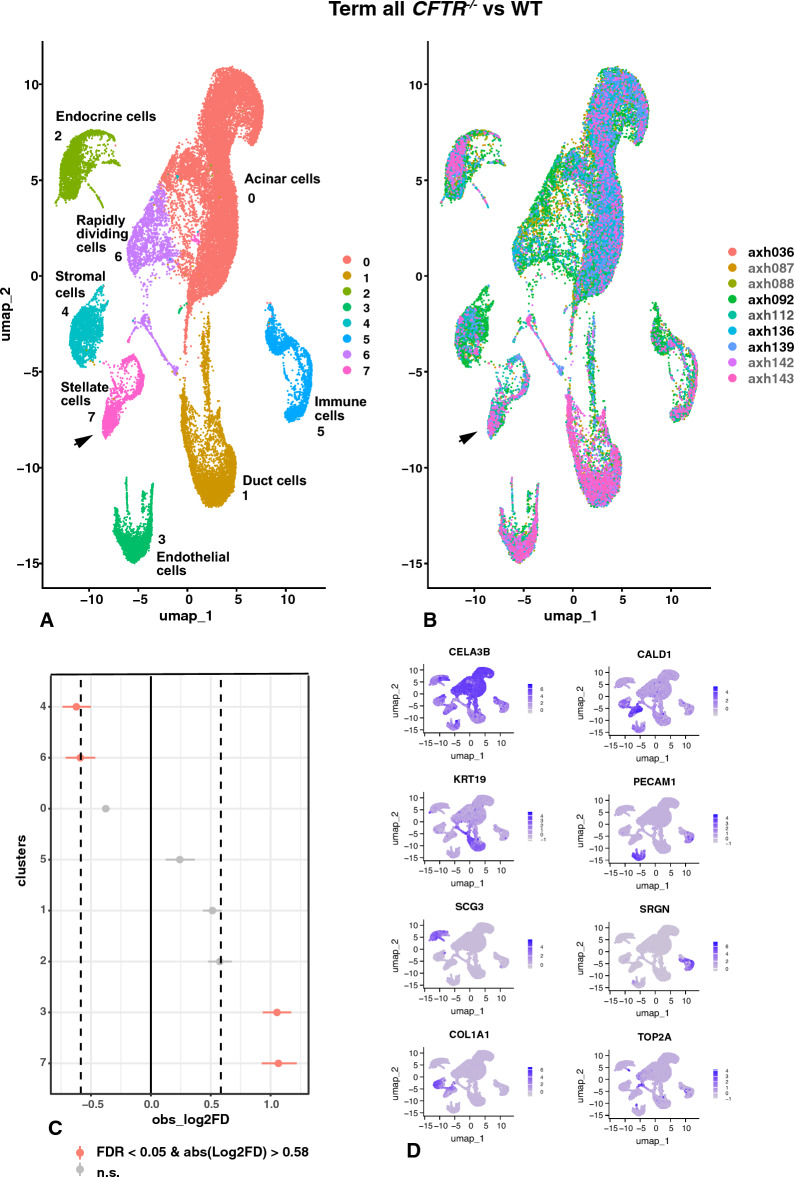
Single-cell RNA-seq defines cell types in term WT and *CFTR*^−/−^ sheep pancreas and shows an activated stellate cell/myofibroblast population is significantly overrepresented in the *CFTR*^−/−^ pancreas. UMAP plots of merged WT (AXH036, AXH092, AXH136, AXH139) and *CFTR*^−/−^ (AXH087, AXH088, AXH112, AXH142, AXH143) sheep pancreas by differential gene expression profiles (**A**) or donor genotype (B, black font WT,  grey font *CFTR*^−/−^). Eight cell clusters are seen in (**A**, **B**) including acinar cells, duct cells, endocrine cells, stromal cells, endothelial cells, activated stellate cells/myofibroblasts (cluster 7, arrowed) and rapidly diving cells. Marker gene lists are shown in Table S9. **C** Single cell proportion test comparing *CFTR*^−/−^ with WT shows significant over representation of activated stellate cells/myofibroblasts. **D** Feature plots show cluster marker genes for clusters 0–7

At term the *CFTR*^*−/−*^ pancreas is usually in an advanced state of destruction (Fig. S1B), with normal acini replaced by cystic lesions, dilated acini and ducts and large areas of fibrosis. We performed scRNA-seq on three term pancreas tissue samples obtained from two *CFTR*^−/−^ lambs produced by natural breeding (AXH087 and AXH088 (2 independent samples from the same lamb) and AXH112), a total of 18,886 cells, one cloned *CFTR*^−/−^ animal (AXH142 and AXH143, two independent samples from the same lamb) with 6,954 cells annotated, and one sample from each of 4 WT sheep (AXH036, AXH092, AXH136 and AXH139) with a total of 21,951 cells annotated). Identity by cluster and by donor UMAPs are shown in Fig. 8A, B, respectively. Eight clusters were identified with marker genes shown in Table S9. Cluster 0 are acinar cells/glandular epithelial cells with markers of zymogen granule enzymes such as *CELA3B* (Fig. 8D), *CPA1*, colipase (*CLPS*), *PLA2G1B*, *PRSS1*, *DNASE1,* chymotrypsin C (*CTRC), SPINK1*, chymotrypsinogen B1 (*CTRB1*), *CEL*, *CELA1,* and syncollin (*SYCN*), which encodes a secretory granule membrane protein involved in zymogen granule fusion (Edwardson et al. 1997), and also *AQP8.* Cluster 1 are duct epithelial cells with marker genes including *SLC4A4*, *KRT19* (Fig. 8D), *CLND4, EHF*, *KRT8*, and *CFTR*. Endocrine cells are found in cluster 2, with *TTR, SCG5, SCG3* (Fig. 8D) and *CHGA* at the top of the marker gene list together with *ISL1*, though the cells are not distinguished by endocrine cell sub-type. Cluster 3 cells show classical markers of endothelial cells based upon *CLEC3B, PECAM1* (Fig. 8D), *PLVAP*, *VWF* and *PTPRB.* Cluster 4 are stromal cells/fibroblasts identified by differential expression of *COL6A*1, *COL1A1* (Fig. 8D)*, COL1A2*, *COL3A1*, *COL6A3* together with *DCN, LUM* and *VIM.* At least 2 types of immune cells are included in cluster 5, with 2 merging subclusters, both of which show *SRGN* (Fig. 8D), *FCER1G, CD53*, BCL2 related protein A1 (*BCL2A1*), protein tyrosine phosphatase receptor type C (*PTPRC*), which is a key regulator of B- and T-cell antigen receptor signaling, and *NFKB1*, among many other immune function-related genes in the marker list. The marker gene list for cluster 6 is indicative of rapidly dividing cells, with differential expression of cell cycle genes such as aurora kinase A (*AURKA*), cell division cycle 20 (*CDC20*), the microtubule destabilizing protein stathmin (*STMN1*), *CENPW*, *CENPF* and *TOP2A* (Fig. 8D). Of note rapidly dividing cells are also seen in WT pancreas at 80- and 120-days gestation (Figs. 6A, 7A) and are not overrepresented in the *CFTR*^*−/−*^ pancreas (Fig. 8C). Cluster 7 (marked by the arrow in Fig. 8A) are another population of stromal cells with differentially expressed marker genes including *CALD1* (Fig. 8D), *TPM2, TAGLN* and *SPARC*. This cluster may include both quiescent and activated stellate cells since in addition to the marker genes *COL4A1/2*, *COL6A1* and *COL3A1*, both *ACTA2* and *PMP22* are highly expressed. Together, the profile of the term *CFTR*^*−/−*^ sheep pancreas shown by scRNA-seq reveals a damaged organ, consistent with the histology shown in Fig. S1B. A comparison of cell contributions to each cluster (sc cell proportions test) from WT and *CFTR*^*−/−*^ animals (Fig. 8C) suggests that cells from *CFTR*^*−/−*^animals are significantly underrepresented in cluster 4 (stromal cells) and cluster 6 (rapidly dividing cells) and significantly overrepresented in clusters 3 (endothelial), and particularly cluster 7 (stellate cells). The difference in distributions of stromal cell types from individual WT and *CFTR*^*−/−*^ animals can be seen in Fig. 8B (and Fig. S13C) and cluster 7 appears show a transition from quiescent to activated stellate cells. Though such comparisons should be interpreted with caution on a relatively small number of animals, the results suggest that at term, the shift from stromal cells to stellate cells may reflect the extensive fibrotic reaction seen in the *CFTR*^*−/−*^ pancreas.

### Validation of scRNA-seq observations by immunostaining

**Fig. 9:**
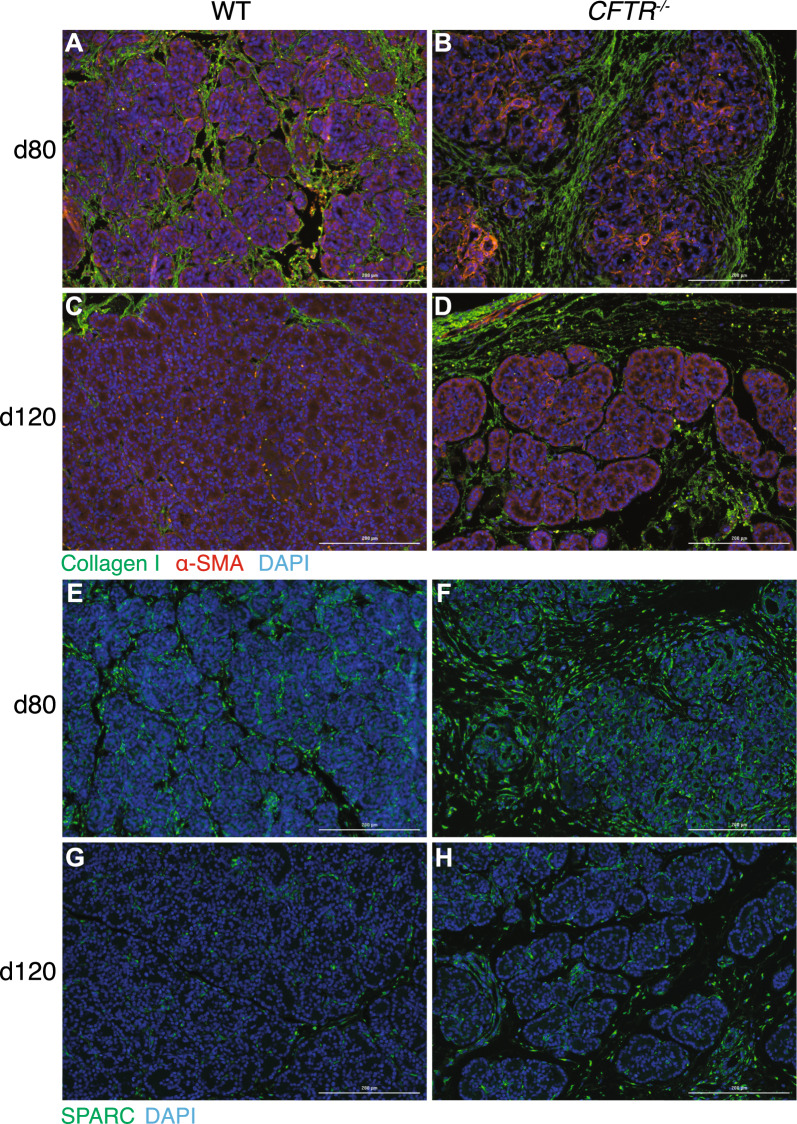
Elevated expression of stellate cell markers in *CFTR*^−/−^ pancreas compared to WT at 80- and 120-days gestation. Tissue sections are stained with antibodies specific for (**A**–**D**) type-I collagen (COL1A1, green) and α smooth muscle actin (ACTA2, red), and (**E**–**H**) secreted protein acidic and cysteine rich (SPARC, green) wild-type (**A**, **C**, **E**, **G**) and *CFTR*^*−/−*^ pancreas. Nuclei were counterstained with 4’,6-diamidino-2-phenylindole (DAPI). COL1A1 and SPARC staining and αSMA-positive expressing cells were detected in the interlobular spaces of the WT (left panels: **A**, **C**) and *CFTR*^−/−^ (right panels: **B**, **D**) pancreas at 80-days (**A**, **B**, **D**, **F**) and 120 days of gestation (**C**, **D**, **G**, **H**). Size bar = 200 μm for all panels

Tissue sections from pancreas samples taken at the same gestational ages as the bulk RNA-seq, though generally not from the same fetuses (only 65- and 100-day WT and 100-day *CFTR*^*−/−*^ were the same), were used for immunostaining. More extensive areas of collagen I-staining were seen in the *CFTR*^*−/−*^ pancreas compared to WT at all time points, particularly in interstitial tissue between pancreatic lobules (Fig. 9A–D) in green; A, C, (WT), B, D (*CFTR*^*−/−*^); 80-days (A, B) and 120-days (C, D); and Fig. S8, all timepoints). Although already evident at day 65 and 80 these *CFTR*^*−/−*^-associated changes become more apparent at later time points. Concurrently, abundance of a smooth muscle actin, which is encoded by a top marker gene for PSCs, is increased in the *CFTR*^*−/−*^ pancreas (Fig. 9A–D) in red; (Fig. S8 all timepoints) as is the frequency of SPARC-positive cells especially at 65- and 80 days (Fig. 9E–H) in green; E, G, (WT), F, H, (*CFTR*^*−/−*^) (Fig. S9 all timepoints). We also stained tissue sections with an antibody specific for APOD as a marker of the activated stellate cell population, however, though APOD-positive cells were observed in the interlobular spaces, due to the rarity of the cell population it was not possible to make significant comparisons between WT and *CFTR*^*−/−*^ tissues (Fig. S10). Lastly, to further investigate the identity of a subcluster of cells in the term pancreas samples from some donors (not evident in the combined analysis in Fig. 8), which in addition to showing characteristic acinar cell marker genes also had features of stressed or apoptotic cells, we performed cleaved caspase-3 and TUNEL assays on pancreas tissue samples throughout gestation. No significant increase in cleaved caspase 3 staining was observed between WT and *CFTR*^*−/−*^ pancreas at all-time points, suggesting apoptosis was not a key mechanism. However, a significant increase in TUNEL staining was seen in the *CFTR*^*−/−*^ pancreas compared to WT at day 120 gestation and term animals, suggestive of DNA-damage (Fig. S11). The TUNEL staining we observe apparently in mesenchymal cells in the *CFTR*^*−/−*^ animals is not seen prior to day 120 and is thought not to be background staining, but instead is likely indicative of necrosis. In recent work on mechanisms of kidney injury (Moore et al. 2021), a similar pattern of irregular TUNEL-positive staining in tissue areas with little DAPI staining was thought to show leakage of DNA fragments from damaged cells, consistent with cell death by necrosis.

### Pseudotime analysis of sheep pancreas scRNA-seq data

**Fig. 10:**
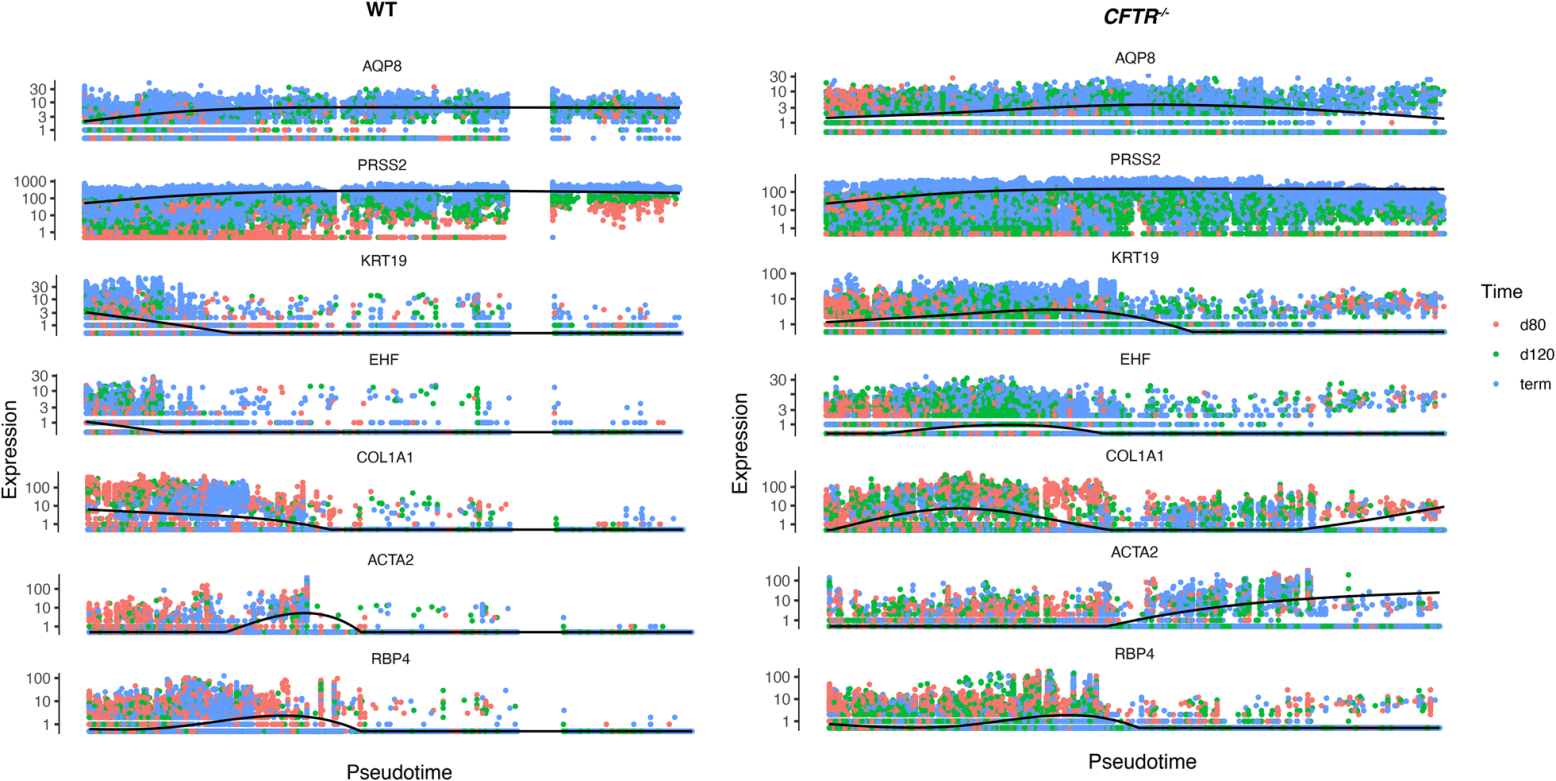
Pseudotime analysis of scRNA-seq data using Monocle 3 shows altered gene expression profiles in WT and *CFTR*^−/−^ pancreatic duct and stromal cells. Markers for acinar cells *AQP8* and *PRSS2*; for duct cells *KRT19*, and *EHF*; *COL1A1* for stromal cells and *ACTA2* and *RBP4* for activated and quiescent stellate cells

Even though we have real-time gene expression data through sheep gestation, it was of interest to perform pseudotime analysis of these data to measure the progress of individual cells through pancreas development and compare cells from WT and *CFTR*^*−/−*^ animals. For this analysis we used Monocle 3 and chose marker genes for each cell cluster defined at 80 days gestation above to construct single cell trajectories. These included: *AQP8* and *PRSS2* for acinar cells; *KRT19* and *EHF* for duct cells; *COL1A1* for stromal cells and *RBP4* and *ACTA2* for stellate cells (all shown Fig. 10); and *SCGN*, *TTR* and *ISL1* for endocrine cells (Fig. S12).

Of most interest are the genes identifying acinar cells, duct cells and stellate cells, which show different trajectories in WT and *CFTR*^*−/−*^ pancreas (Fig. 10) and are consistent with our interpretation of data from the bulk RNA-seq and scRNA-seq above. Considering the splines (black lines) in Fig. 10: acinar cell markers appear to show similar profiles in the *CFTR*^*−/−*^ cells compared to WT, though AQP8 declines towards later pseudotime; duct cell markers, which decline to very low levels early in pseudotime in WT cells show prolonged expression through pseudotime in *CFTR*^*−/−*^ cells, consistent with the observed overrepresentation of duct cells in *CFTR*^*−/−*^ pancreas in the single cell proportion test at 80 days (Fig. 6C); while stellate cells marked by *ACTA2* appear to show increased expression later in pseudotime only in *CFTR*^*−/−*^ pancreas, in accordance with the overrepresentation of stellate cells in *CFTR*^*−/−*^ pancreas observed in the single cell proportion test at term (Fig. 8C). No clear differences were seen in the splines of endocrine markers in WT and *CFTR*^*−/−*^ samples (Fig. S12).

### Comparison of cloned and naturally bred* CFTR*^*−/−*^animals through gestation

In our previous work defining the pathology of *CFTR*^*−/−*^ in sheep we focused on cloned animals as these were the only available models (Fan et al. 2018; Van Wettere et al. 2022; Kerschner et al. 2023; Viotti Perisse et al. 2021). Over the last two sheep breeding seasons we also generated *CFTR*^*−/−*^ lambs by natural breeding of heterozygous (*CFTR*^±^) ewes and rams. The majority of term animals analyzed here were naturally bred with only one being a cloned *CFTR*^*−/−*^ animal (Table 1). Moreover, we also optimized amniocentesis on sheep at 55 + 3 days gestation to enable in utero genotyping of fetuses and collected twin naturally bred *CFTR*^*−/−*^ lambs at 120 days gestation. The genotype was reconfirmed after collection. This enabled a preliminary comparison between cloned and non-cloned *CFTR*^*−/−*^ animals, though data should be interpreted with caution on n = 2 non-cloned tissue samples (Table 1), particularly since the *CFTR*^*−/−*^-associated pancreatic lesions at 120 days are focal, with some areas of the tissue apparently normal and others showing significant pathology (Van Wettere et al. 2022). The data for the 120 days samples shown in Fig. 7 only include cloned animals. When comparing all *CFTR*^*−/−*^ samples (cloned and non-cloned) with WT at 120 days the unique activated stellate cells/injured Schwann cell cluster (8) was still the only overrepresented cell type in the single cell proportion test, though there were fewer of these cells in the non-cloned animals (data not shown) suggesting a more severe phenotype in the cloned *CFTR*^*−/−*^ lambs*.* However, when comparing the two naturally bred *CFTR*^*−/−*^ animals with WT, the single cell proportion test was greatly skewed by fewer erythroid cells in the *CFTR*^*−/−*^ samples. This was likely an artefact reflecting varying efficacy of the red blood cell removal step in the protocol (data not shown), making further interpretation more challenging.

## Discussion

Utilizing a sheep model of cystic fibrosis has enabled us to examine the initiation and progression of CF pancreatic disease in utero with transcriptomic precision at both whole tissue and single cell levels. Previous studies detected *CFTR* mRNA in pancreatic ducts in early human gestation (Hyde et al. 1997; Foulkes and Harris 1993), so an early phenotype resulting from loss of CFTR was not unexpected. However, the recent suggestion that loss of CFTR might impair tubulogenesis (Breton et al. 2016) raised doubt over the initiating events in CF pancreatic pathology. Here we describe a fibrotic and damage response by 80 days gestation (~ 21 weeks in human) and the presence of a unique population of cells that may be activated stellate cells or injured Schwann cells, which are significantly more abundant in *CFTR*^*−/−*^ animals than in WT at 80- and 120 days gestation. The observations of a very early (by 65 days of gestation) and enduring fibrotic response in *CFTR*^*−/−*^ animals were confirmed by immunohistology using antibodies specific for collagen 1A (COL1A1), which is produced by all stromal cells, actin alpha 2, smooth muscle actin (ACTA2), which is a marker of activated stellate cells/ myofibroblasts in the liver (Hoffmann et al. 2020; de Smet et al. 2021) and secreted protein acidic and cysteine rich (SPARC), (Fig. 9; Figs. S8, S9). By 65 days of sheep gestation (~ 16 weeks in human) an abundance of stromal cells (green, collagen 1A) is evident in the *CFTR*^*−/−*^ fetal pancreas compared to WT, and distension of acini, with evidence for stellate cell (red, alpha smooth muscle actin) activation/ proliferation also seen (Fig. 9A, B). As gestation proceeds (Fig. S8C, D, 80 days, Fig. S8G, H, 120 days) the stromal cell involvement proceeds in parallel with the loss of acinar tissue and stellate cells are prominent. At term parts of the pancreas are already destroyed (Fig. S8I, J). It is notable that though we observed an under representation of acinar cells in the scRNA-seq data from 80 day *CFTR*^*−/−*^ pancreas compared to WT, this was not recapitulated in the term *CFTR*^*−/−*^ pancreas samples, despite their altered histology. One possible explanation is the difficulty of capturing live acinar cells in the single cell preparation protocols since these cells readily autolyze post-mortem. Perhaps we are only capturing a sub-population of more resilient acinar cells (for example those close to the ductal tree or adjacent to the stromal cells surrounding the acinar bundles) in both WT and *CFTR*^*−/−*^ pancreas samples. Alternatively, the results may simply reflect sampling location since the acinar loss is multifocal and variable between *CFTR*^*−/−*^ animals.

Our data suggest that CF pancreatic pathology is probably not due to a failure to generate the ductal tree but instead is a reaction to the abnormal ductal microenvironment in the absence of functional CFTR, as originally suggested. Loss of CFTR-mediated chloride and bicarbonate secretion (Gray et al. 1990; Saint-Criq and Gray 2017) is accompanied by a lack of hydration of the pancreatic duct epithelium. This in turn likely causes the deposition of secreted protein on the duct epithelium, initial intralobular duct obstruction (we reported mucus accumulation in ducts and acini of *CFTR*^*−/−*^ sheep fetal pancreas previously (Van Wettere et al. 2022)) and tissue damage resulting in activation of stellate cells and fibrosis. The stellate cell activation may also have an immunological/inflammatory component. Pancreatic duct obstruction appears to be the main cause of acinar loss, as enzymes released from zymogen granules cannot reach the duodenum and instead cause cell necrosis. Fibrosis is likely a later event in the disease course. PSCs may be progenitors for several cell types in the pancreas. They normally proliferate and migrate through the pancreatic tissue where they synthesize ECM proteins and also matrix degrading enzymes (for example MMPs and TIMPs). In healthy pancreas, PSCs regulate normal ECM processing. In contrast, during pancreatic injury PSC activation can disrupt the fine-tuned balance between ECM production and remodeling, causing fibrosis and other phenotypes that are consistent with pancreatic pathology in *CFTR*^*−/−*^ sheep.

It is of interest that activated hepatic stellate cells (HSCs) were implicated in the etiology of cystic-fibrosis-associated liver disease (CFLD) in an earlier study of human liver biopsies (Lewindon et al. 2002). Cells identified by colocalization of collagen 1A1 mRNA (by in situ hybridization) and alpha smooth muscle actin (by immunostaining), suggesting activated HSCs/myofibroblast, were shown to be the source of excess collagen production in a person with CF and hepatic fibrosis. Moreover, bile duct epithelial cells were suggested to be the major source of transforming growth factor beta (TGFB) which may stimulate collagen synthesis by HSCs in CF (Lewindon et al. 2002). In the sheep pancreas at 80 days gestation the TGFB subunits are marker genes for stromal cells and myofibroblasts and are not apparently differentially expressed in pancreatic duct epithelial cells, suggesting fibrosis may be a later event. However, it is possible that a specific sub-type of ductal epithelial cell is important in TGFB activation of stellate cells and that we have not reached sufficient single cell resolution to identify it. The origins of duct cells in the pancreas were broadly understood (Edlund 2002), though more recent scRNA-seq analysis has revealed additional complexity (Qadir et al. 2020). Furthermore, recent bulk and scRNA-seq analysis of hepatic stellate cell activation suggests that transcriptional programs driving the initiation of HSCs remain active in chronic liver disease (de Smet et al. 2021). A similar persistence of multiple activated stellate cell types in the *CFTR*^*−/−*^ sheep pancreas may underlie our observations. The similarity of many marker genes in activated stellate cells and injured Schwann cells makes it challenging to definitively assign a cell identity to the unique cluster of cells seen to be overrepresented at 80- and 120-days gestation in *CFTR*^*−/−*^ animals. Indeed, UMAP plots for several key marker genes show that expression is rarely limited to a single differentiated cell population at these gestational ages (Fig. S13A, B). If indeed these are Schwann cells, the relative lack of expression of genes encoding myelin proteins indicates that they are likely either dedifferentiating or damaged, possibly as a direct response to the pancreatic enzyme induced acinar autolysis and associated fibrotic reaction in the *CFTR*^*−/−*^ pancreas.

An earlier detailed analysis of post-natal human and mouse pancreas by scRNA-seq identified multiple stellate cell types at different stages of activation, and also Schwann cell dedifferentiation in the pancreas (Baron et al. 2016). Though not all the critical markers of these cells are annotated in the sheep genome these data are helpful in interpreting the scRNA-seq data presented here. The definition of different clusters in our data is also more challenging as we are looking at cell populations that are differentiating through organ development. At 80 days we see platelet derived growth factor receptor alpha (*PDGFRA*) as a marker gene only for cluster 2 (stromal cells), along with *COL4A1* which is a marker of quiescent and activated stellate cells. *COL1A1* and *LUM* are marker genes for cluster 2 though are seen in activated stellate cells in the earlier human data (Baron et al. 2016). TIMP metallopeptidase inhibitor 1 (*TIMP1*), another marker of activated stellate cells is seen in both clusters 2 and 8 (stellate cells), while *SPARC* is in the marker gene list for both clusters 2 and 6 (activated stellate cells/injured Schwann cells) and 8 (Fig. S13A). Our particular interest in cluster 6, due to its significant overrepresentation in the cloned *CFTR*^*−/−*^ animals, led us to speculate that though these cells show many markers of activated stellate cells they are perhaps more similar to cells of neural crest origin, specifically injured or dedifferentiated Schwann cells without myelin (e.g., myelin basic protein, *MBP*). *S100B*, *NGFR* and *PLP1* are all markers for cluster 6, while *CRYAB* is a marker for cluster 8 (though is expressed in many cell types at 80 days gestation, Fig. S13A) and *PMP22* is seen in clusters 2, 6 and 8. Most notably, *ID4* and *FOXD3*, which are transcription factors associated with Schwann cell dedifferentiation (Baron et al. 2016), and endothelin receptor type B (*EDNRB*) and nestin (*NES*) both encoding proteins associated with this process are also in the marker gene list for cluster 6. Though in the earlier work it was suggested that this cluster might have dedifferentiated during extraction and culture, it is likely that in the *CFTR*^*−/−*^ pancreas these cells are overrepresented due to disease -associated organ damage in the tissue. In contrast at 120 days cluster 8, which appears to have a similar identity to cluster 6 at 80 days, is also overrepresented in the *CFTR*^*−/−*^ pancreas but has a less clear identity. Though *CRYAB* and *PLP1* are marker genes (Fig. S13B), *NGFR* and *S100B* are not (the latter is a marker of duct cells at this gestational age). *ID4* is a marker of clusters 5 (stellate cells) and 8 as are *EDNRB* and *NES* perhaps suggesting a closer relationship of this cluster to activated stellate cells than injured Schwann cells. This might also correlate with the observed overrepresentation of quiescent- and activated stellate cells in the *CFTR*^*−/−*^ pancreas at term.

Comparative genomics studies can throw light on important aspects of human disease, so we next considered earlier work on the development of pancreatic disease in pig and ferret models of CF. In the CF pig, pancreatic tissue was collected from 8 fetuses in utero, during one time window (83–90 days out of the total 114-day gestation period), RNA was extracted and used for microarray studies of gene expression (Abu-El-Haija et al. 2012). This time-period is broadly equivalent to ~ 100–110 days gestation in sheep, where term is at ~ 147 days.

Gene set enrichment analysis (GSEA) at this single timepoint identified elevated response to injury, inflammatory response and host defense response, innate immunity, complement cascade, tissue remodeling and fibroblast proliferation, in addition to induction of apoptosis, as enriched processes in the CF fetuses. Focusing on specific genes that contributed to these pathways, both *ACTA2* and *TGFB1* showed increased expression in fetal and newborn CF pig pancreas, indicative of activation of fibrosis. Furthermore, proinflammatory, complement cascade and proapoptotic genes were all upregulated and thus implicated in contributing to pancreatic destruction. When compared to bulk RNA-seq data from the *CFTR*^*−/−*^ sheep pancreas reported here, we did not observe consistent increases in *CXCL8* or *NFKB1* transcripts in utero and newborn, although *TGFB1* was elevated in *CFTR*^*−/−*^ sheep fetuses at 65 days (Fig. S6E–G). Also, complement activation as measured by elevated transcripts of *C3*, *C1Q* and *CFB*, were seen in CF pig but not in *CFTR*^*−/−*^ sheep. It is however, of interest that in the scRNA-seq data at 80 days gestation we see a significant increase in one immune cell cluster (10) in the *CFTR*^*−/−*^ fetuses. It is notable that although the data from the CF pig pancreas were interpreted to support plugging and obstruction of the pancreatic ducts triggering an inflammatory response that ultimately leads to tissue destruction, overt fibrosis was not seen until weeks to months after birth. In contrast, as in humans with CF, fibrosis is evident in the *CFTR*^*−/−*^ sheep in utero and the results presented here suggest that stellate cell activation by mechanisms other than profound inflammation and complement activation initiates the process. Further, these events start to happen very early, within the first half of gestation in *CFTR*^*−/−*^ sheep and pwCF.

Next comparing pancreatic disease in the CF ferret (Rotti et al. 2018) with our observations presented here in the *CFTR*^*−/−*^ sheep, the most notable difference is the time scale, as the ferret pathology primarily appears at term and develops after birth. Moreover, there is a remarkable replacement of acinar tissue by fat/adipose tissue in the CF ferret, which is not seen in the *CFTR*^*−/−*^ sheep pancreas at term. Three stages of CF pancreatic exocrine disease in ferrets are defined: (I) inflammation, (II) associated stellate cell activation followed by replacement of exocrine tissue with adipose tissue and fibrosis, which progresses in stage III. Thus, the CF ferret pancreatic pathology appears not to be fully consistent with our observation in the *CFTR*^*−/−*^ sheep, where acinar tissue is not replaced by fat, but rather acinar loss and stromal collapse results in cystic spaces, with some multifocal fibrosis, consistent with the early disease course in humans.

Lastly, in the initial description of the *CFTR*^*−/−*^ sheep, we observed pancreatic hypoplasia in some animals, which was thought to be a cloning-related phenomenon (Fan et al. 2018). The *CFTR*^*−/−*^ animals generated by cloning over several subsequent sheep breeding seasons and used here to examine the developmental time course (day 65- day 120) of CF (Van Wettere et al. 2022) did not exhibit such profound pancreatic hypoplasia. Moreover, all but one of the *CFTR*^*−/−*^ animals investigated here at term were not cloned, but rather the result of natural breeding of heterozygote *CFTR*^±^ ewes and rams, with their genotype confirmed within ~ 6 h of birth. Hence, the observation of overrepresentation of quiescent and activated stellate cells at term is not an artifact of cloning procedures but rather a specific feature of the *CFTR*^*−/−*^ sheep pancreas. Given the major focus on PSCs in the context of developing novel therapeutic approached to pancreas cancer it is encouraging to speculate that advances in this field may have applications to early attenuation of pancreatic disease in pwCF.

### Supplementary Information

Supplementary Material 1.

Supplementary Material 2.

Supplementary Material 3.

Supplementary Material 4.

Supplementary Material 5.

Supplementary Material 6.

Supplementary Material 7.

Supplementary Material 8.

Supplementary Material 9.

Supplementary Material 10.

Supplementary Material 11.

Supplementary Material 12.

## Data Availability

All raw and processed deep sequence data are deposited at GEO: GSE254376. There are no other materials to make available.

## References

[CR1] Abi-Nader KN, Boyd M, Flake AW, Mehta V, Peebles D, David AL. Animal models for prenatal gene therapy: the sheep model. Methods Mol Biol. 2012;891:219–48. 10.1007/978-1-61779-873-3_11. 22648775 10.1007/978-1-61779-873-3_11

[CR2] Abu-El-Haija M, Ramachandran S, Meyerholz DK, et al. Pancreatic damage in fetal and newborn cystic fibrosis pigs involves the activation of inflammatory and remodeling pathways. Am J Pathol. 2012;181(2):499–507. 10.1016/j.ajpath.2012.04.024. 22683312 10.1016/j.ajpath.2012.04.024 PMC3409440

[CR3] Alcorn DG, Adamson TM, Maloney JE, Robinson PM. A morphologic and morphometric analysis of fetal lung development in the sheep. Anat Rec. 1981;201(4):655–67. 10.1002/ar.1092010410. 7340570 10.1002/ar.1092010410

[CR4] Andersen DH. Cystic fibrosis of the pancreas and its relation to celiac disease—a clinical and pathological study. Am J Dis Child. 1938;56(2):344–99. 10.1001/archpedi.1938.01980140114013

[CR5] Andersen DH. Cystic fibrosis of the pancreas. J Chronic Dis. 1958;7(1):58–90. 10.1016/0021-9681(58)90185-1. 13491678 10.1016/0021-9681(58)90185-1

[CR6] Apte MV, Haber PS, Applegate TL, et al. Periacinar stellate shaped cells in rat pancreas: identification, isolation, and culture. Gut. 1998;43(1):128–33. 10.1136/gut.43.1.128. 9771417 10.1136/gut.43.1.128 PMC1727174

[CR7] Apte MV, Wilson JS, Lugea A, Pandol SJ. A starring role for stellate cells in the pancreatic cancer microenvironment. Gastroenterology. 2013;144(6):1210–9. 10.1053/j.gastro.2012.11.037. 23622130 10.1053/j.gastro.2012.11.037 PMC3729446

[CR8] Arsenijevic T, Perret J, Van Laethem JL, Delporte C. Aquaporins involvement in pancreas physiology and in pancreatic diseases. Int J Mol Sci. 2019;20(20):5052. 10.3390/ijms20205052. 31614661 10.3390/ijms20205052 PMC6834120

[CR9] Bachem MG, Schneider E, Gross H, et al. Identification, culture, and characterization of pancreatic stellate cells in rats and humans. Gastroenterology. 1998;115(2):421–32. 10.1016/s0016-5085(98)70209-4. 9679048 10.1016/s0016-5085(98)70209-4

[CR10] Bale S, Verma P, Varga J, Bhattacharyya S. Extracellular matrix-derived damage-associated molecular patterns: implications in systemic sclerosis and fibrosis. J Invest Dermatol. 2023. 10.1016/j.jid.2023.04.030. 37452808 10.1016/j.jid.2023.04.030 PMC11974346

[CR11] Banstola A, Reynolds JNJ. The sheep as a large animal model for the investigation and treatment of human disorders. Biology (basel). 2022;11(9):1251. 10.3390/biology11091251. 36138730 10.3390/biology11091251 PMC9495394

[CR12] Baron M, Veres A, Wolock SL, et al. A single-cell transcriptomic map of the human and mouse pancreas reveals inter- and intra-cell population structure. Cell Syst. 2016;3(4):346-360 e4. 10.1016/j.cels.2016.08.011. 27667365 10.1016/j.cels.2016.08.011 PMC5228327

[CR13] Barry JS, Anthony RV. The pregnant sheep as a model for human pregnancy. Theriogenology. 2008;69(1):55–67. 10.1016/j.theriogenology.2007.09.021. 17976713 10.1016/j.theriogenology.2007.09.021 PMC2262949

[CR14] Blouw B, Seals DF, Pass I, Diaz B, Courtneidge SA. A role for the podosome/invadopodia scaffold protein Tks5 in tumor growth in vivo. Eur J Cell Biol. 2008;87(8–9):555–67. 10.1016/j.ejcb.2008.02.008. 18417249 10.1016/j.ejcb.2008.02.008 PMC2629379

[CR15] Boue A, Muller F, Nezelof C, et al. Prenatal diagnosis in 200 pregnancies with a 1-in-4 risk of cystic fibrosis. Hum Genet. 1986;74(3):288–97. 3536726 10.1007/BF00282551

[CR16] Breton S, Ruan YC, Park YJ, Kim B. Regulation of epithelial function, differentiation, and remodeling in the epididymis. Asian J Androl Jan-Feb. 2016;18(1):3–9. 10.4103/1008-682X.165946. 10.4103/1008-682X.165946 PMC4736353 26585699

[CR17] Chandra R, Liddle RA. Recent advances in the regulation of pancreatic secretion. Curr Opin Gastroenterol. 2014;30(5):490–4. 10.1097/MOG.0000000000000099. 25003603 10.1097/MOG.0000000000000099 PMC4229368

[CR18] Chen J, Fu R, Cui Y, et al. LIM-homeodomain transcription factor Isl-1 mediates the effect of leptin on insulin secretion in mice. J Biol Chem. 2013;288(17):12395–405. 10.1074/jbc.M113.450536. 23504315 10.1074/jbc.M113.450536 PMC3636923

[CR19] Chen Y, Lun AT, Smyth GK. From reads to genes to pathways: differential expression analysis of RNA-Seq experiments using Rsubread and the edgeR quasi-likelihood pipeline. F1000 Res. 2016;5:1438. 10.12688/f1000research.8987.2. 10.12688/f1000research.8987.2 PMC4934518 27508061

[CR20] da Huang W, Sherman BT, Lempicki RA. Systematic and integrative analysis of large gene lists using DAVID bioinformatics resources. Nat Protoc. 2009;4(1):44–57. 10.1038/nprot.2008.211. 19131956 10.1038/nprot.2008.211

[CR21] Davies CJ, Fan Z, Morgado KP, et al. Development and characterization of type I interferon receptor knockout sheep: a model for viral immunology and reproductive signaling. Front Genet. 2022;13: 986316. 10.3389/fgene.2022.986316. 36246651 10.3389/fgene.2022.986316 PMC9556006

[CR22] De Smet V, Eysackers N, Merens V, et al. Initiation of hepatic stellate cell activation extends into chronic liver disease. Cell Death Dis. 2021;12(12):1110. 10.1038/s41419-021-04377-1. 34839349 10.1038/s41419-021-04377-1 PMC8627507

[CR23] Dobin A, Davis CA, Schlesinger F, et al. STAR: ultrafast universal RNA-seq aligner. Bioinformatics. 2013;29(1):15–21. 10.1093/bioinformatics/bts635. 23104886 10.1093/bioinformatics/bts635 PMC3530905

[CR24] Dupre J, Ross SA, Watson D, Brown JC. Stimulation of insulin secretion by gastric inhibitory polypeptide in man. J Clin Endocrinol Metab. 1973;37(5):826–8. 10.1210/jcem-37-5-826. 4749457 10.1210/jcem-37-5-826

[CR25] Edlund H. Pancreatic organogenesis-developmental mechanisms and implications for therapy. Nat Rev Genet. 2002;3(7):524–32. 10.1038/nrg841. 12094230 10.1038/nrg841

[CR26] Edwardson JM, An S, Jahn R. The secretory granule protein syncollin binds to syntaxin in a Ca2(+)-sensitive manner. Cell. 1997;90(2):325–33. 10.1016/s0092-8674(00)80340-2. 9244306 10.1016/s0092-8674(00)80340-2

[CR27] Fan Z, Viotti Perisse I, Cotton CU, et al. A sheep model of cystic fibrosis generated by CRISPR/Cas9 disruption of the CFTR gene. JCI Insight. 2018;3(19):e123529. 10.1172/jci.insight.123529. 30282831 10.1172/jci.insight.123529 PMC6237476

[CR28] Foulkes AG, Harris A. Localization of expression of the cystic fibrosis gene in human pancreatic development. Pancreas. 1993;8(1):3–6. 10.1097/00006676-199301000-00003. 7678325 10.1097/00006676-199301000-00003

[CR29] Fournet JC, Junien C. The genetics of neonatal hyperinsulinism. Horm Res. 2003;59(Suppl 1):30–4. 10.1159/000067842. 12566718 10.1159/000067842

[CR30] Froeling FE, Feig C, Chelala C, et al. Retinoic acid-induced pancreatic stellate cell quiescence reduces paracrine Wnt-beta-catenin signaling to slow tumor progression. Gastroenterology. 2011;141(4):1486–97. 10.1053/j.gastro.2011.06.047. 21704588 10.1053/j.gastro.2011.06.047

[CR31] Graff SM, Johnson SR, Leo PJ, et al. A KCNK16 mutation causing TALK-1 gain of function is associated with maturity-onset diabetes of the young. JCI Insight. 2021;6(13):e138057. 10.1172/jci.insight.138057. 34032641 10.1172/jci.insight.138057 PMC8410089

[CR32] Gray MA, Pollard CE, Harris A, Coleman L, Greenwell JR, Argent BE. Anion selectivity and block of the small-conductance chloride channel on pancreatic duct cells. Am J Physiol. 1990;259(5 Pt 1):C752–61. 1700622 10.1152/ajpcell.1990.259.5.C752

[CR33] Greene LJ, Pubols MH, Bartelt DC. Human pancreatic secretory trypsin inhibitor. Methods Enzymol. 1976;45:813–25. 10.1016/s0076-6879(76)45075-9. 1012034 10.1016/s0076-6879(76)45075-9

[CR34] Gremlich S, Porret A, Hani EH, et al. Cloning, functional expression, and chromosomal localization of the human pancreatic islet glucose-dependent insulinotropic polypeptide receptor. Diabetes. 1995;44(10):1202–8. 10.2337/diab.44.10.1202. 7556958 10.2337/diab.44.10.1202

[CR35] Han J, Kang D, Kim D. Functional properties of four splice variants of a human pancreatic tandem-pore K+ channel, TALK-1. Am J Physiol Cell Physiol. 2003;285(3):C529–38. 10.1152/ajpcell.00601.2002. 12724142 10.1152/ajpcell.00601.2002

[CR36] Hao Y, Hao S, Andersen-Nissen E, et al. Integrated analysis of multimodal single-cell data. Cell. 2021;184(13):3573-3587 e29. 10.1016/j.cell.2021.04.048. 34062119 10.1016/j.cell.2021.04.048 PMC8238499

[CR37] Harris A. Human molecular genetics and the long road to treating cystic fibrosis. Hum Mol Genet. 2021;30(R2):R264–73. 10.1093/hmg/ddab191. 34245257 10.1093/hmg/ddab191 PMC8490019

[CR38] Herold Z, Doleschall M, Somogyi A. Role and function of granin proteins in diabetes mellitus. World J Diabetes. 2021;12(7):1081–92. 10.4239/wjd.v12.i7.1081. 34326956 10.4239/wjd.v12.i7.1081 PMC8311481

[CR39] Hirano K, Tada M, Isayama H, et al. High alcohol consumption increases the risk of pancreatic stone formation and pancreatic atrophy in autoimmune pancreatitis. Pancreas. 2013;42(3):502–5. 10.1097/MPA.0b013e31826b3984. 23146923 10.1097/MPA.0b013e31826b3984

[CR40] Hoffmann C, Djerir NEH, Danckaert A, et al. Hepatic stellate cell hypertrophy is associated with metabolic liver fibrosis. Sci Rep. 2020;10(1):3850. 10.1038/s41598-020-60615-0. 32123215 10.1038/s41598-020-60615-0 PMC7052210

[CR41] Hrabak P, Kalousova M, Krechler T, Zima T. Pancreatic stellate cells—rising stars in pancreatic pathologies. Physiol Res. 2021;70(Suppl 4):S597–616. 10.33549/physiolres.934783. 35199546 10.33549/physiolres.934783 PMC9054180

[CR42] Hyde K, Reid CJ, Tebbutt SJ, Weide L, Hollingsworth MA, Harris A. The cystic fibrosis transmembrane conductance regulator as a marker of human pancreatic duct development. Gastroenterology. 1997;113(3):914–9. 9287984 10.1016/S0016-5085(97)70187-2

[CR43] Ikejiri N. The vitamin A-storing cells in the human and rat pancreas. Kurume Med J. 1990;37(2):67–81. 10.2739/kurumemedj.37.67. 2255178 10.2739/kurumemedj.37.67

[CR44] Kerschner JL, Paranjapye A, Schacht M, et al. Transcriptomic analysis of lung development in wildtype and CFTR^(−/−)^ sheep suggests an early inflammatory signature in the CF distal lung. Funct Integr Genomics. 2023;23(2):135. 10.1007/s10142-023-01050-y. 37085733 10.1007/s10142-023-01050-y PMC10121546

[CR45] Lewindon PJ, Pereira TN, Hoskins AC, et al. The role of hepatic stellate cells and transforming growth factor-beta(1) in cystic fibrosis liver disease. Am J Pathol. 2002;160(5):1705–15. 10.1016/s0002-9440(10)61117-0. 12000722 10.1016/s0002-9440(10)61117-0 PMC1850885

[CR46] Li XY, Zhai WJ, Teng CB. Notch signaling in pancreatic development. Int J Mol Sci. 2015;17(1):48. 10.3390/ijms17010048. 26729103 10.3390/ijms17010048 PMC4730293

[CR47] Liao Y, Smyth GK, Shi W. featureCounts: an efficient general purpose program for assigning sequence reads to genomic features. Bioinformatics. 2014;30(7):923–30. 10.1093/bioinformatics/btt656. 24227677 10.1093/bioinformatics/btt656

[CR48] Lovatt D, Tamburino A, Krasowska-Zoladek A, et al. scRNA-seq generates a molecular map of emerging cell subtypes after sciatic nerve injury in rats. Commun Biol. 2022;5(1):1105. 10.1038/s42003-022-03970-0. 36261573 10.1038/s42003-022-03970-0 PMC9581950

[CR49] Marino CR, Matovcik LM, Gorelick FS, Cohn JA. Localization of the cystic fibrosis transmembrane conductance regulator in pancreas. J Clin Invest. 1991;88(2):712–6. 1713921 10.1172/JCI115358 PMC295422

[CR50] Masamune A, Shimosegawa T. Pancreatic stellate cells—multi-functional cells in the pancreas. Pancreatology. 2013;13(2):102–5. 10.1016/j.pan.2012.12.058. 23561965 10.1016/j.pan.2012.12.058

[CR51] Moore CL, Savenka AV, Basnakian AG. TUNEL assay: a powerful tool for kidney injury evaluation. Int J Mol Sci. 2021;22(1):412. 10.3390/ijms22010412. 33401733 10.3390/ijms22010412 PMC7795088

[CR52] Murray ER, Menezes S, Henry JC, et al. Disruption of pancreatic stellate cell myofibroblast phenotype promotes pancreatic tumor invasion. Cell Rep. 2022;38(4):110227. 10.1016/j.celrep.2021.110227. 35081338 10.1016/j.celrep.2021.110227 PMC8810397

[CR53] Napolitano T, Silvano S, Vieira A, et al. Role of ghrelin in pancreatic development and function. Diabetes Obes Metab. 2018;20(Suppl 2):3–10. 10.1111/dom.13385. 30230184 10.1111/dom.13385

[CR54] Olaniru OE, Kadolsky U, Kannambath S, et al. Single-cell transcriptomic and spatial landscapes of the developing human pancreas. Cell Metab. 2023;35(1):184-199 e5. 10.1016/j.cmet.2022.11.009. 36513063 10.1016/j.cmet.2022.11.009

[CR55] Paranjapye A, Leir SH, Huang F, Kerschner JL, Harris A. Cell function and identity revealed by comparative scRNA-seq analysis in human nasal, bronchial and epididymis epithelia. Eur J Cell Biol. 2022;101(3): 151231. 10.1016/j.ejcb.2022.151231. 35597096 10.1016/j.ejcb.2022.151231 PMC9357053

[CR56] Pickard MR, Mourtada-Maarabouni M, Williams GT. Candidate tumour suppressor Fau regulates apoptosis in human cells: an essential role for Bcl-G. Biochim Biophys Acta. 2011;1812(9):1146–53. 10.1016/j.bbadis.2011.04.009. 21550398 10.1016/j.bbadis.2011.04.009

[CR57] Qadir MMF, Alvarez-Cubela S, Klein D, et al. Single-cell resolution analysis of the human pancreatic ductal progenitor cell niche. Proc Natl Acad Sci USA. 2020;117(20):10876–87. 10.1073/pnas.1918314117. 32354994 10.1073/pnas.1918314117 PMC7245071

[CR58] Raudvere U, Kolberg L, Kuzmin I, et al. g:Profiler: a web server for functional enrichment analysis and conversions of gene lists (2019 update). Nucleic Acids Res. 2019;47(W1):W191–8. 10.1093/nar/gkz369. 31066453 10.1093/nar/gkz369 PMC6602461

[CR59] Reid CJ, Hyde K, Ho SB, Harris A. Cystic fibrosis of the pancreas: involvement of MUC6 mucin in obstruction of pancreatic ducts. Mol Med. 1997;3(6):403–11. 9234245 10.1007/BF03401687 PMC2230211

[CR60] Reimand J, Arak T, Adler P, et al. g:Profiler—a web server for functional interpretation of gene lists (2016 update). Nucleic Acids Res. 2016. 10.1093/nar/gkw199. 27098042 10.1093/nar/gkw199 PMC4987867

[CR61] Ricci F, Kern SE, Hruban RH, Iacobuzio-Donahue CA. Stromal responses to carcinomas of the pancreas: juxtatumoral gene expression conforms to the infiltrating pattern and not the biologic subtype. Cancer Biol Ther. 2005;4(3):302–7. 10.4161/cbt.4.3.1501. 15876873 10.4161/cbt.4.3.1501

[CR62] Rotti PG, Xie W, Poudel A, et al. Pancreatic and islet remodeling in cystic fibrosis transmembrane conductance regulator (CFTR) knockout ferrets. Am J Pathol. 2018;188(4):876–90. 10.1016/j.ajpath.2017.12.015. 29366680 10.1016/j.ajpath.2017.12.015 PMC5963477

[CR63] Saint-Criq V, Gray MA. Role of CFTR in epithelial physiology. Cell Mol Life Sci. 2017;74(1):93–115. 10.1007/s00018-016-2391-y. 27714410 10.1007/s00018-016-2391-y PMC5209439

[CR64] Sherman MH. Stellate cells in tissue repair, inflammation, and cancer. Annu Rev Cell Dev Biol. 2018;34:333–55. 10.1146/annurev-cellbio-100617-062855. 30028641 10.1146/annurev-cellbio-100617-062855

[CR65] Sherman BT, Hao M, Qiu J, et al. DAVID: a web server for functional enrichment analysis and functional annotation of gene lists (2021 update). Nucleic Acids Res. 2022. 10.1093/nar/gkac194. 35325185 10.1093/nar/gkac194 PMC9252805

[CR66] Van Wettere AJ, Leir SH, Cotton CU, et al. Early developmental phenotypes in the cystic fibrosis sheep model. Faseb Bioadv. 2022. 10.1096/fba.2022-00085. 36643895 10.1096/fba.2022-00085 PMC9832529

[CR67] Viotti Perisse I, Fan Z, Van Wettere A, et al. Sheep models of F508del and G542X cystic fibrosis mutations show cellular responses to human therapeutics. FASEB Bioadv. 2021;3(10):841–54. 10.1096/fba.2021-00043. 34632318 10.1096/fba.2021-00043 PMC8493969

[CR68] Watanabe T, Yonekura H, Terazono K, Yamamoto H, Okamoto H. Complete nucleotide sequence of human reg gene and its expression in normal and tumoral tissues. The reg protein, pancreatic stone protein, and pancreatic thread protein are one and the same product of the gene. J Biol Chem. 1990;265(13):7432–9. 2332435 10.1016/S0021-9258(19)39132-X

[CR69] Watari N, Hotta Y, Mabuchi Y. Morphological studies on a vitamin A-storing cell and its complex with macrophage observed in mouse pancreatic tissues following excess vitamin A administration. Okajimas Folia Anat Jpn. 1982;58(4–6):837–58. 10.2535/ofaj1936.58.4-6_837. 7122019 10.2535/ofaj1936.58.4-6_837

[CR70] Wice BM, Gordon JI. A tetraspan membrane glycoprotein produced in the human intestinal epithelium and liver that can regulate cell density-dependent proliferation. J Biol Chem. 1995;270(37):21907–18. 10.1074/jbc.270.37.21907. 7665614 10.1074/jbc.270.37.21907

[CR71] Wollam J, Mahata S, Riopel M, et al. Chromogranin A regulates vesicle storage and mitochondrial dynamics to influence insulin secretion. Cell Tissue Res. 2017;368(3):487–501. 10.1007/s00441-017-2580-5. 28220294 10.1007/s00441-017-2580-5 PMC10843982

[CR72] Xiang Y, Tan YR, Zhang JS, et al. Wound repair and proliferation of bronchial epithelial cells regulated by CTNNAL1. J Cell Biochem. 2008;103(3):920–30. 10.1002/jcb.21461. 17647259 10.1002/jcb.21461

[CR73] Yang M, Hall J, Fan Z, et al. Oocytes from small and large follicles exhibit similar development competence following goat cloning despite their differences in meiotic and cytoplasmic maturation. Theriogenology. 2016;86(9):2302–11. 10.1016/j.theriogenology.2016.07.026. 27650944 10.1016/j.theriogenology.2016.07.026

[CR74] Yang X, Chen J, Wang J, et al. Very-low-density lipoprotein receptor-enhanced lipid metabolism in pancreatic stellate cells promotes pancreatic fibrosis. Immunity. 2022;55(7):1185-1199 e8. 10.1016/j.immuni.2022.06.001. 35738281 10.1016/j.immuni.2022.06.001

[CR75] Zhang JS, Nelson M, Wang L, et al. Identification and chromosomal localization of CTNNAL1, a novel protein homologous to alpha-catenin. Genomics. 1998;54(1):149–54. 10.1006/geno.1998.5458. 9806841 10.1006/geno.1998.5458

[CR76] Zhang K, Shen X, Wu J, et al. Endoplasmic reticulum stress activates cleavage of CREBH to induce a systemic inflammatory response. Cell. 2006;124(3):587–99. 10.1016/j.cell.2005.11.040. 16469704 10.1016/j.cell.2005.11.040

