# Supplementary material for: Stellate cells are in utero markers of pancreatic disease in cystic fibrosis
Source: Mol Med. 2024 Aug 7;30:115. doi: 10.1186/s10020-024-00871-2 (PMC11304907; doi:10.1186/s10020-024-00871-2)
Supplement: Supplementary file 1 — Supplementary Material 1. [file 10020_2024_871_MOESM1_ESM.pdf]

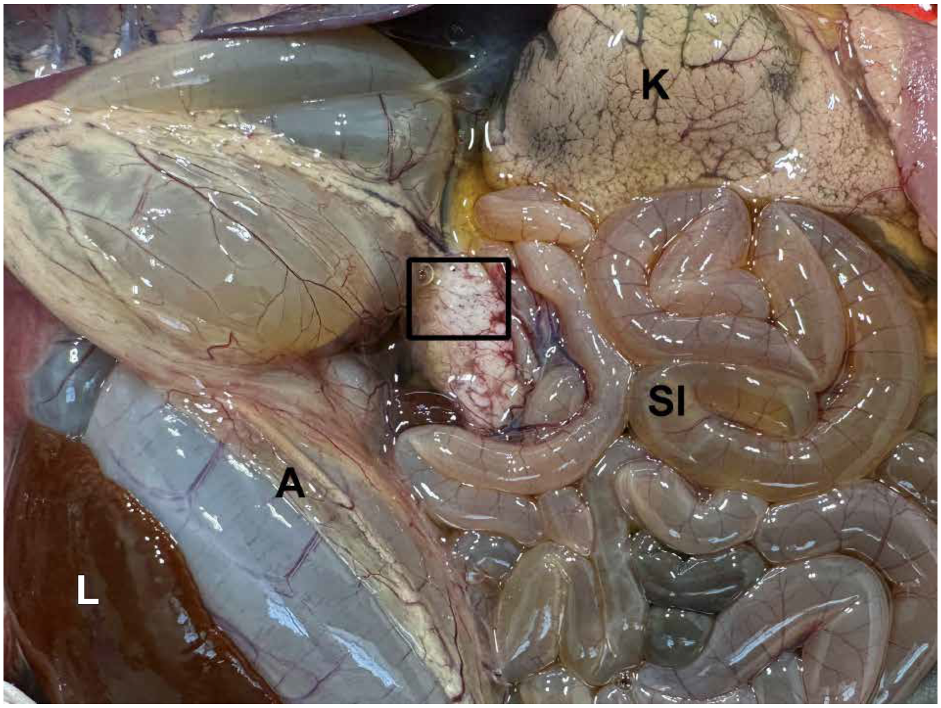

Figure S1A Pancreas of a 120-day-old WT sheep fetus. The square indicate the area of pancreas sampled for RNA extraction. A: abomasum; L: Liver; K: Kidney; SI: Small intestine.

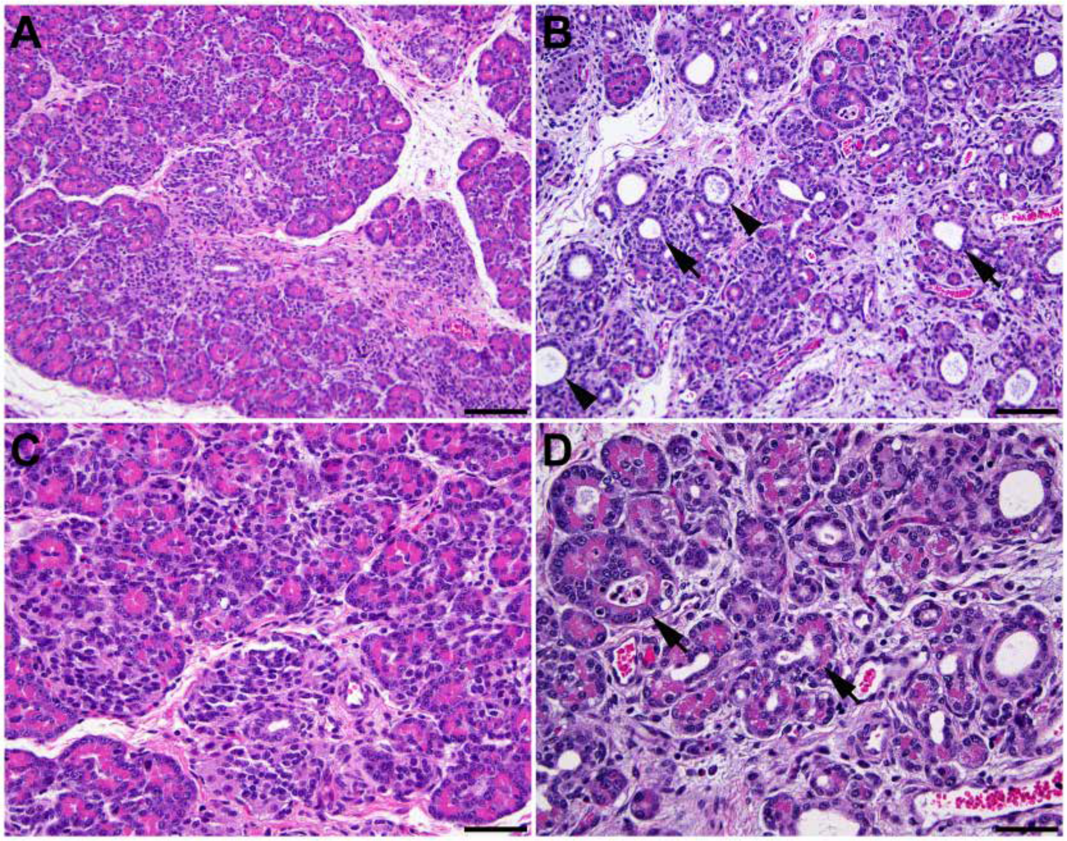

Figure S1B Pancreatic pathology of a newborn *CFTR*$^{-/-}$ (cloned) lamb. (A) and (C) Histology of normal pancreas from a control lamb. (B) and (D) *CFTR*$^{-/-}$ lamb. Pancreatic acinar atrophy with stromal collapse and dilatation of acini are evident (arrow). Mucus is present in some duct lumens (arrowhead). Hematoxylin and eosin staining. (A) and (B) 200×. Bar = 100 µm. (C) and (D) 400×. Bar = 50 µm. Reproduced from *doi: 10.1093/hmg/ddab191.*

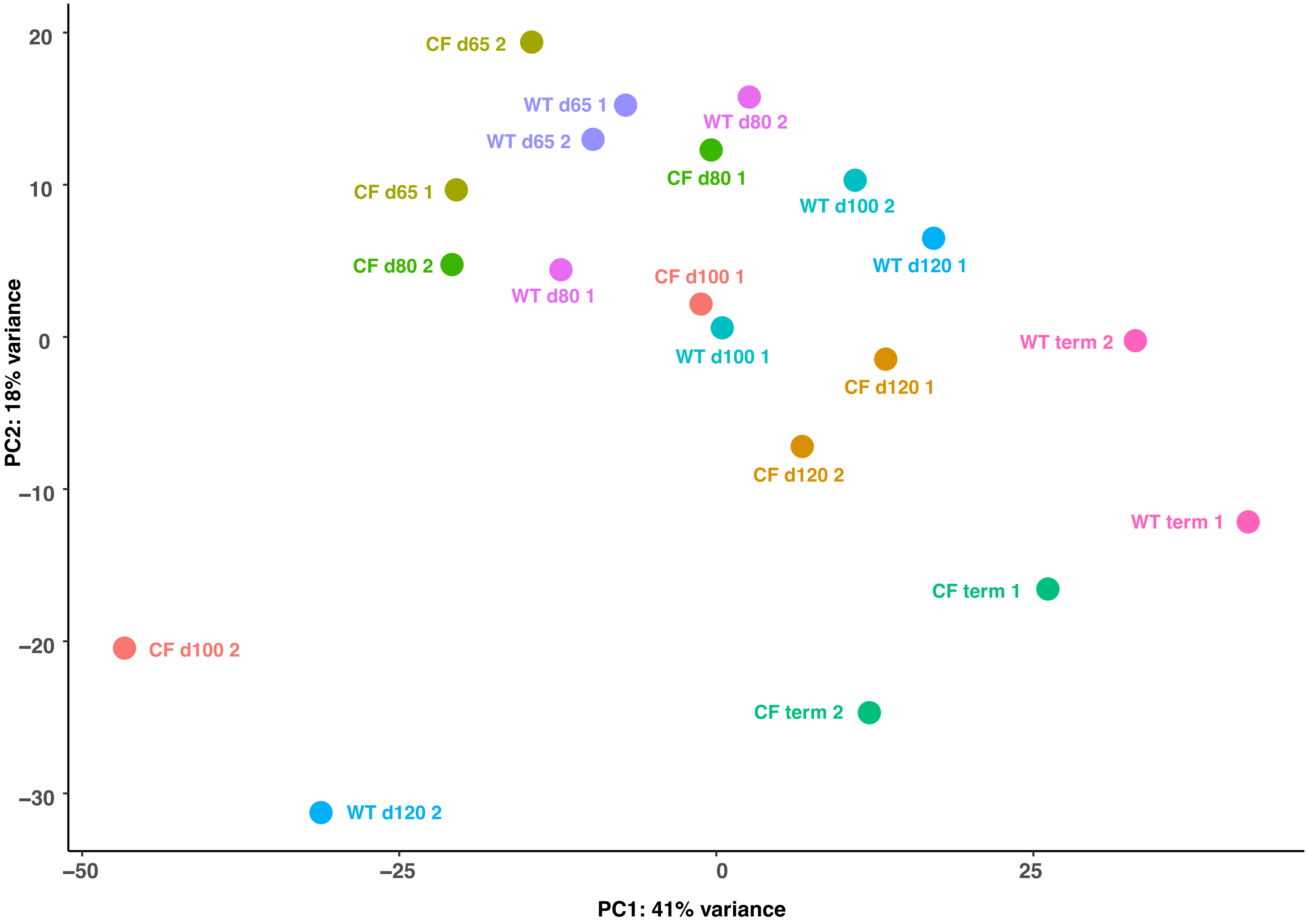

Figure S2 Principal component analysis plots showing RNA-seq sample distribution of WT and *CFTR*$^{-/-}$ (CF) pancreas .

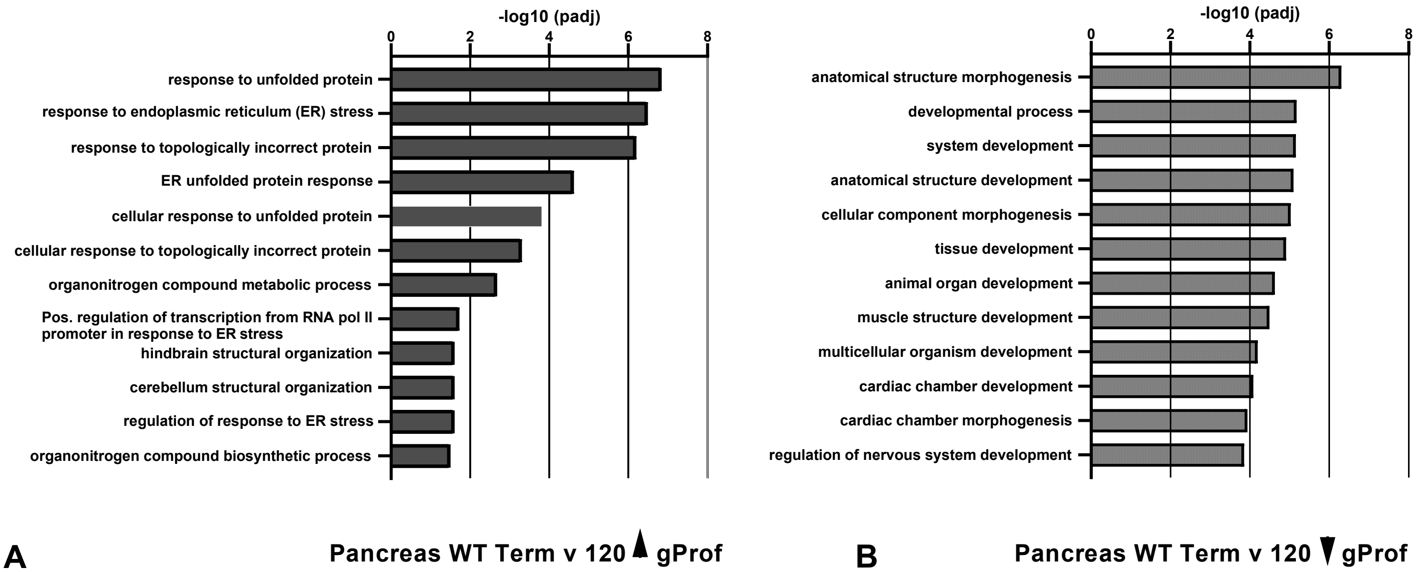

Figure S3 Gene ontology process enrichment analyses of differentially expressed genes between gestational time points in WT sheep pancreas. Differentially expressed genes were filtered to enrich for genes with a fold change ≥ ±1.5 and Benjamini-Hochberg adjusted p-value ≤ 0.01 for 120 day samples and p-value ≤ 0.001 for term samples. Gene ontology analysis by gProfiler and up to the top 12 biological processes (BP) are shown. A) Genes upregulated between 120 days and term. B) Genes downregulated between 120 days and term.

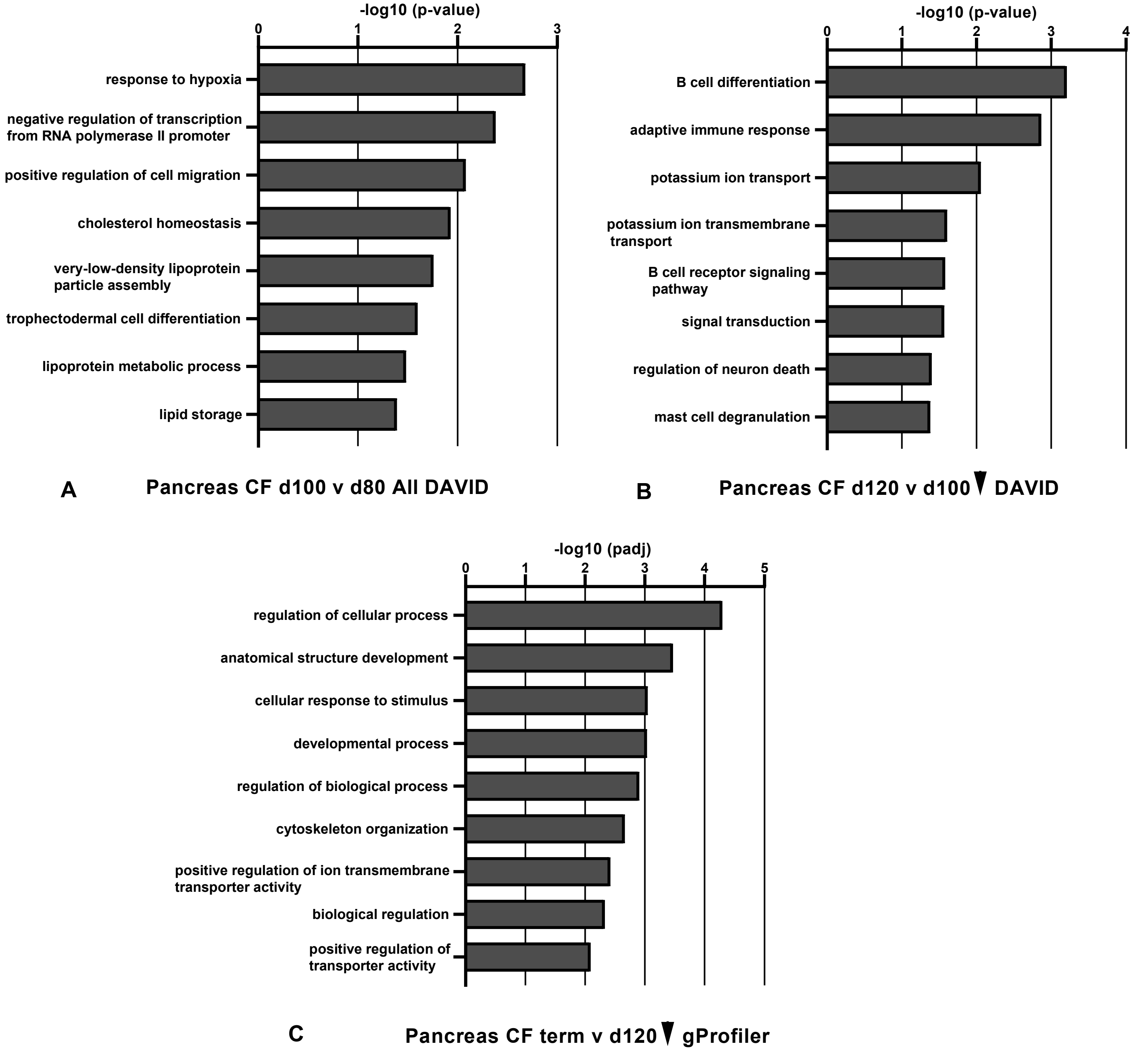

Figure S4. Gene ontology process enrichment analyses of differentially expressed genes between gestational time points in *CFTR*$^{-/-}$ sheep pancreas. Differentially expressed genes were filtered to enrich for genes with a fold change ≥ ±1.5 and Benjamini-Hochberg adjusted p-value ≤ 0.01 for all samples except term for which a p-value ≤ 0.001 was used. Gene ontology analysis by DAVID (A, B) and gProfiler (C) and the top biological processes (BP) are shown. A) DEGs between 80- and 100-days. B) Genes downregulated between 100- and 120-days. (C) Genes downregulated between 120 days and term.

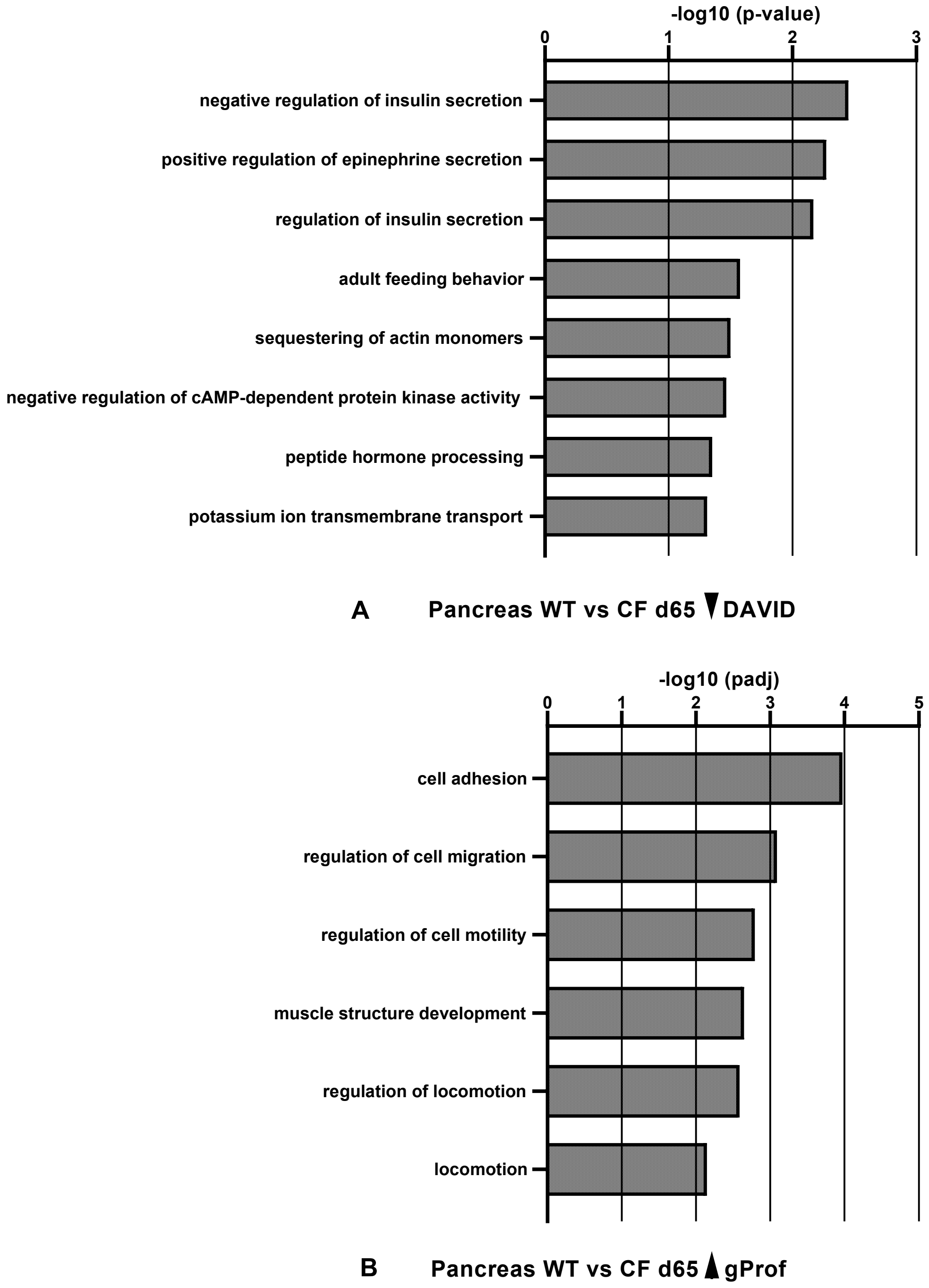

Figure S5 Gene ontology process enrichment analyses of differentially expressed genes between WT and *CFTR*$^{-/-}$ sheep pancreas at 65 days. Differentially expressed genes were filtered to enrich for genes with a fold change ≥ ±1.5 and Benjamini-Hochberg adjusted p-value ≤ 0.01. Gene ontology analysis by DAVID (A) and gProfiler (B) and the top biological processes (BP) are shown. A) Genes downregulated in *CFTR*$^{-/-}$ compared to WT. B) Genes upregulated in *CFTR*$^{-/-}$ compared to WT.

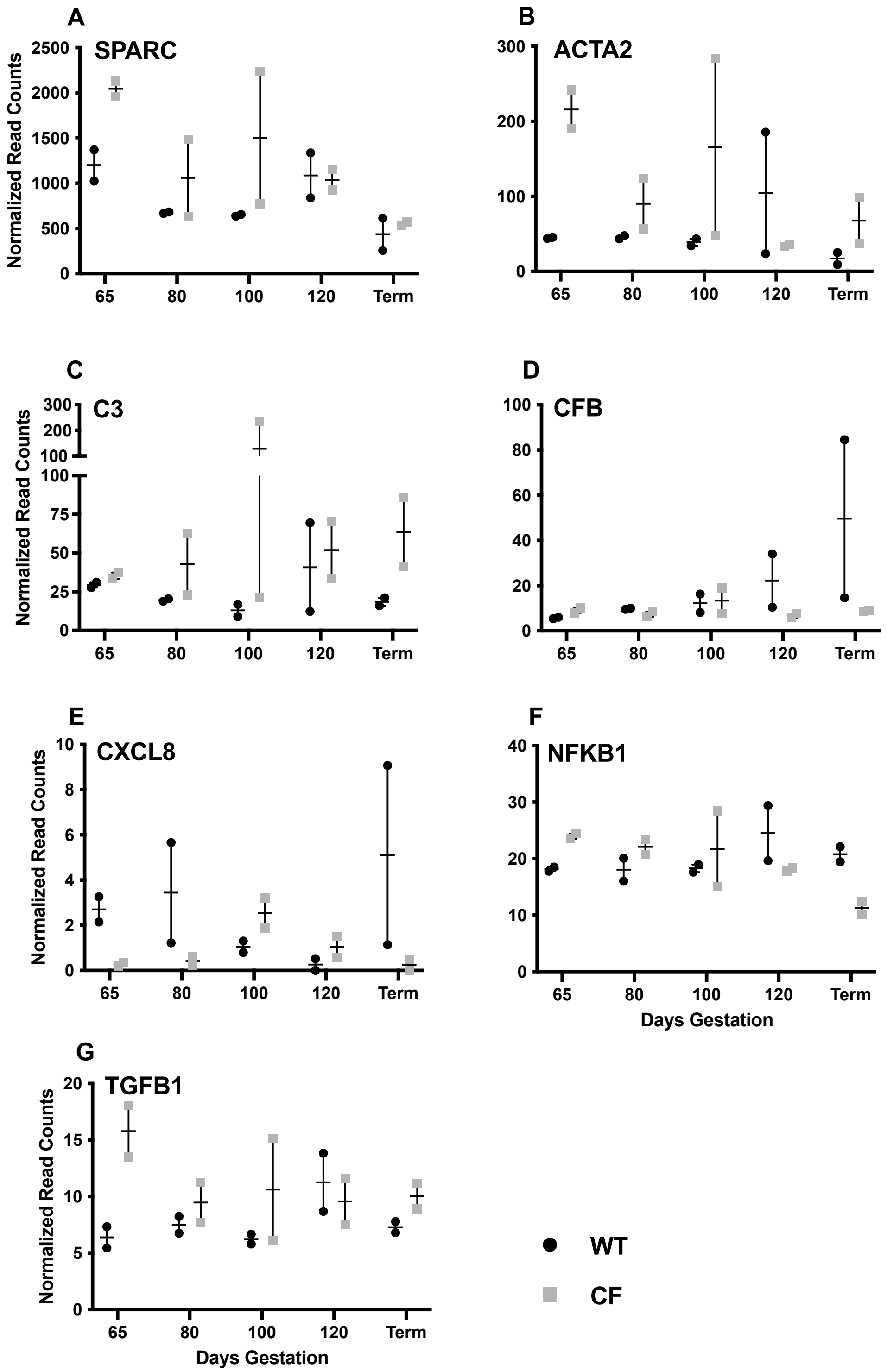

Figure S6 Developmental profile of marker gene expression for stellate cells, inflammation and the complement cascade in WT and CFTR-/- sheep pancreas. Normalized read counts from bulk RNA-seq data show gene expression through gestation at 65-, 80-, 100-, 120-days gestation and at term in WT and CFTR-/- (CF) sheep. (A, B) Stellate cell markers SPARC and ACTA2; (C, D) Complement cascade markers C3 and CFB; (E -G) inflammatory markers: CXCL8, NFKB1, TGFB1. WT values are shown as black circles and CF values as grey squares.

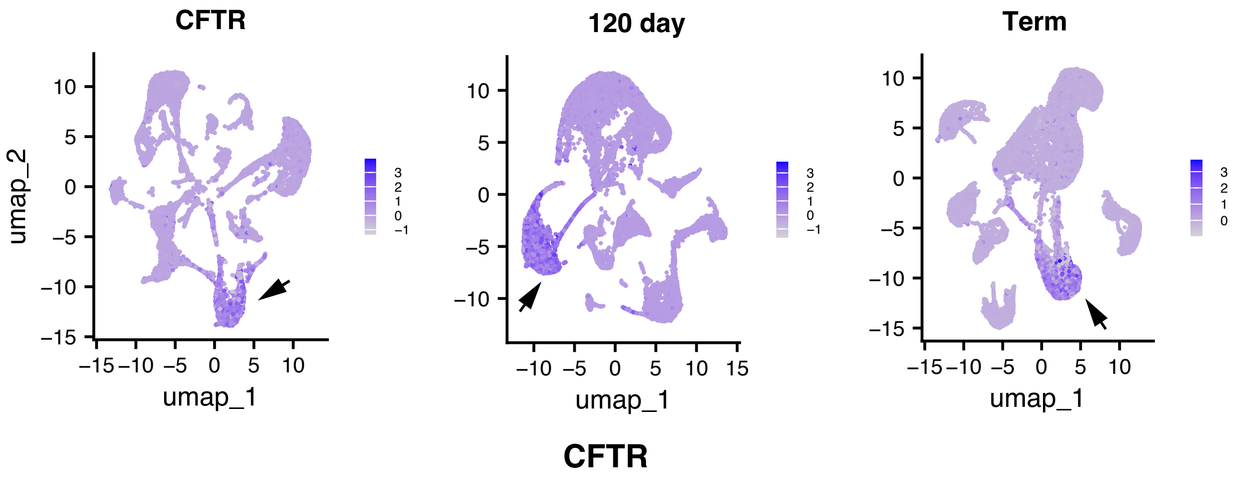

Figure S7. High expression of CFTR in a subpopulation of duct epithelial cells at 80, 120 days and at term. For cluster identities see Fig. 6, 7 and 8.

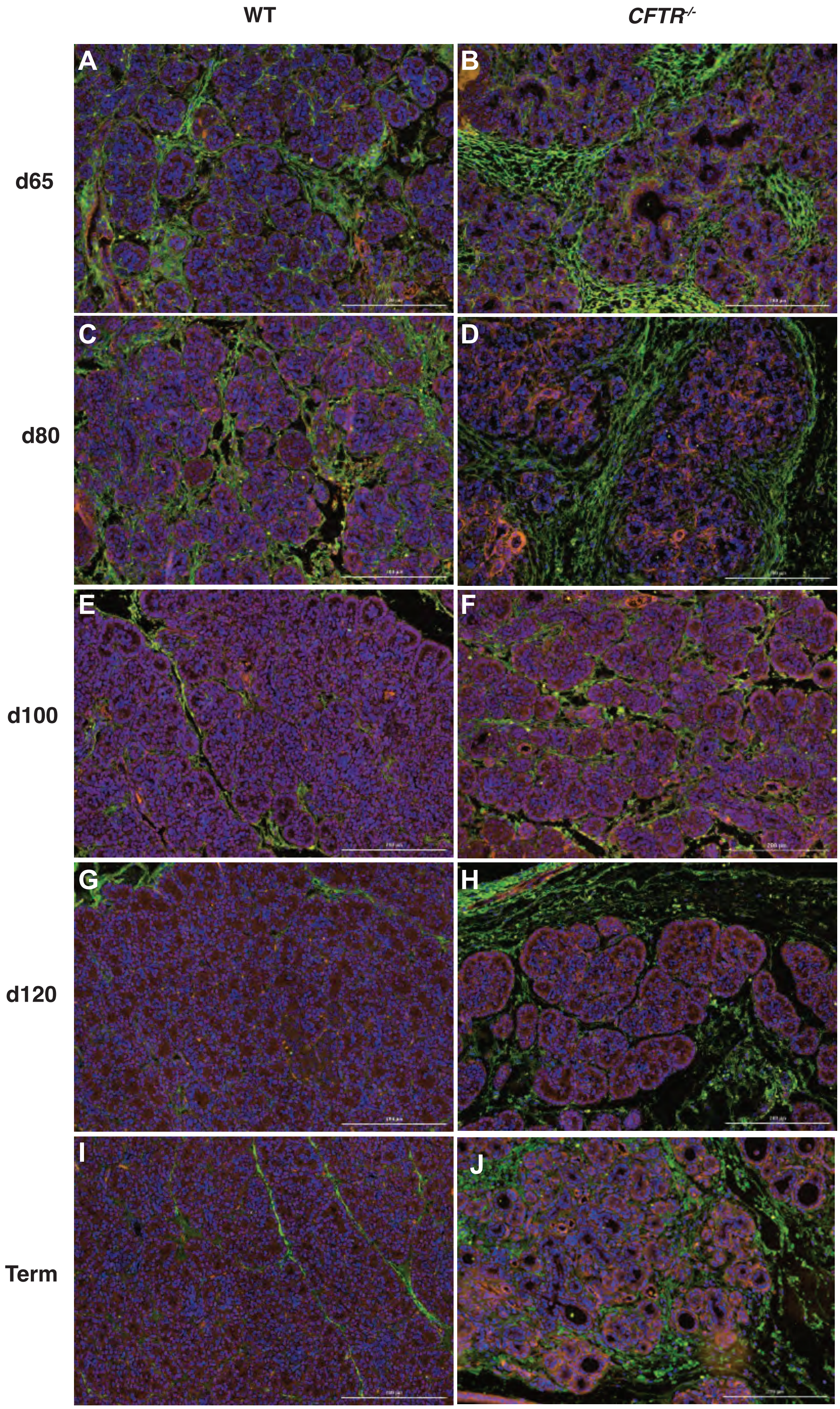

Figure S8 Elevated expression of stellate cell markers in *CFTR*-/- pancreas (right) compared to WT (left) through gestation at 65-, 80-, 100-, 120-days and term. Tissue sections are stained with antibodies specific for type-I collagen (COL1A1, green) and α smooth muscle actin (ACTA2, red). Size bar = 200μm for all panels.

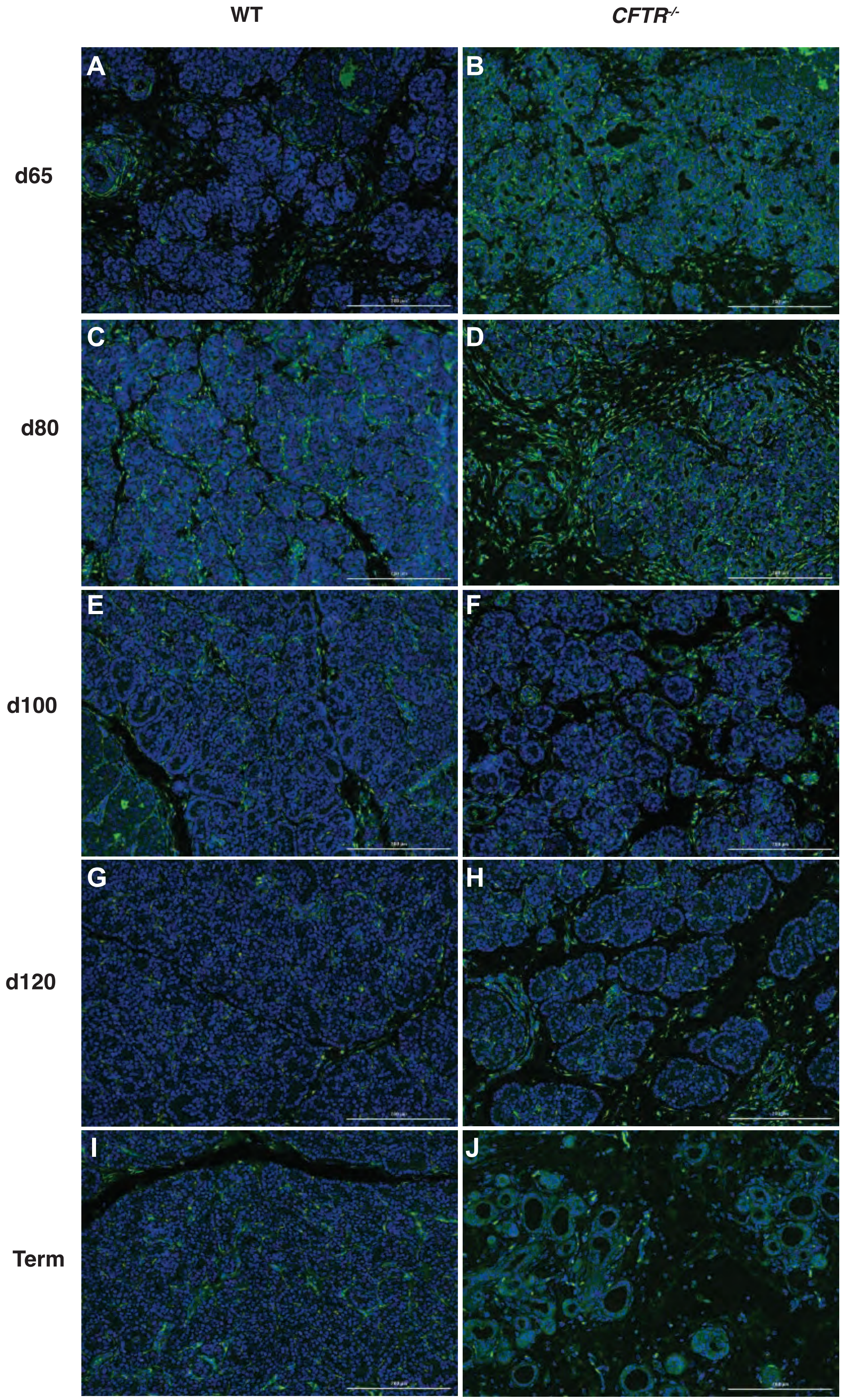

Figure S9 Elevated expression of stellate cell marker SPARC in *CFTR*$^{-/-}$ pancreas (right) compared to WT (left) through gestation at 65-, 80-, 100-, 120-days and term. Tissue sections are stained with an antibody specific for SPARC, green. Size bar = 200μm for all panels.

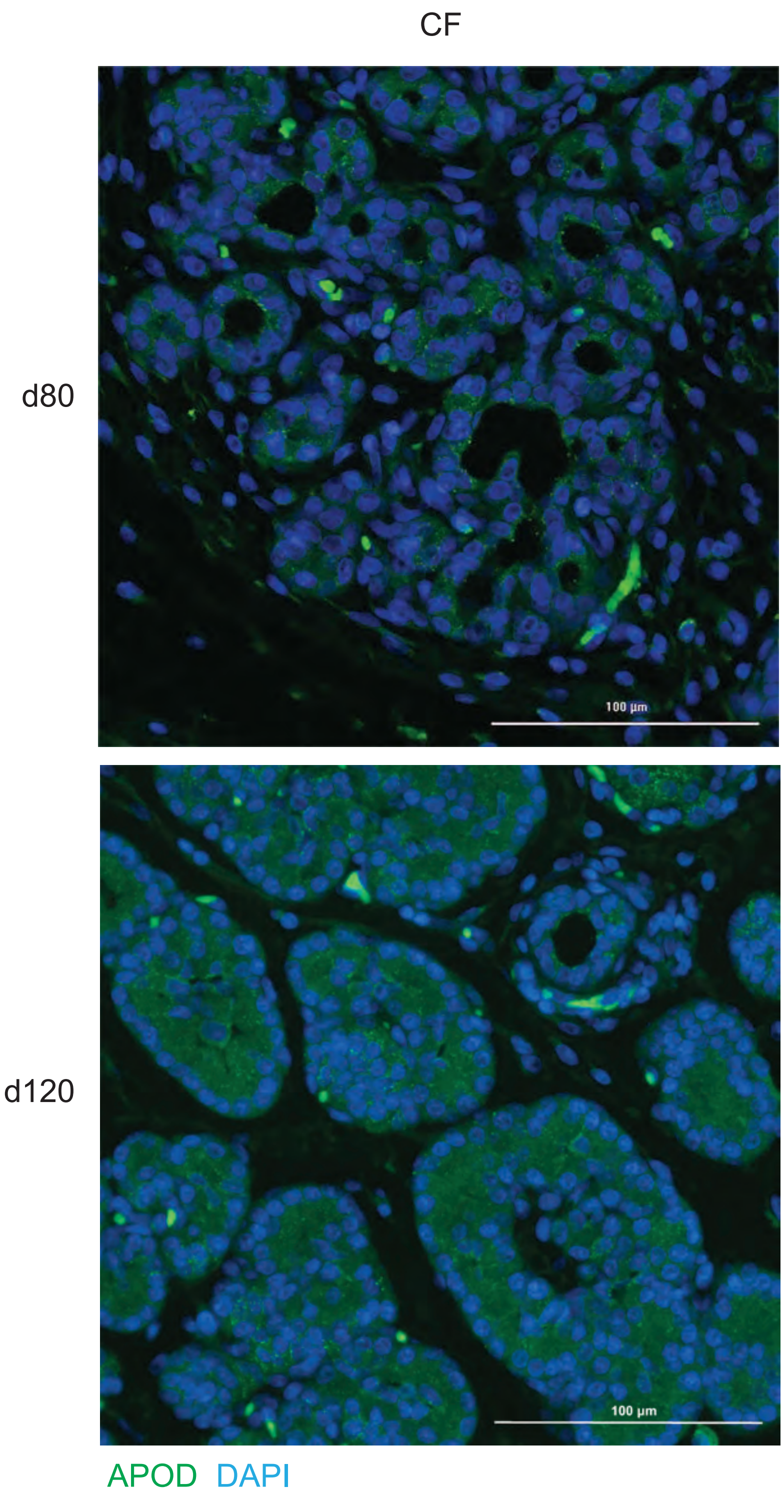

Figure S10

APOD staining in the CF pancreas at 80 days and 120 days. Nuclei are counterstained with DAPI. Size bar = 200µm in all panels.

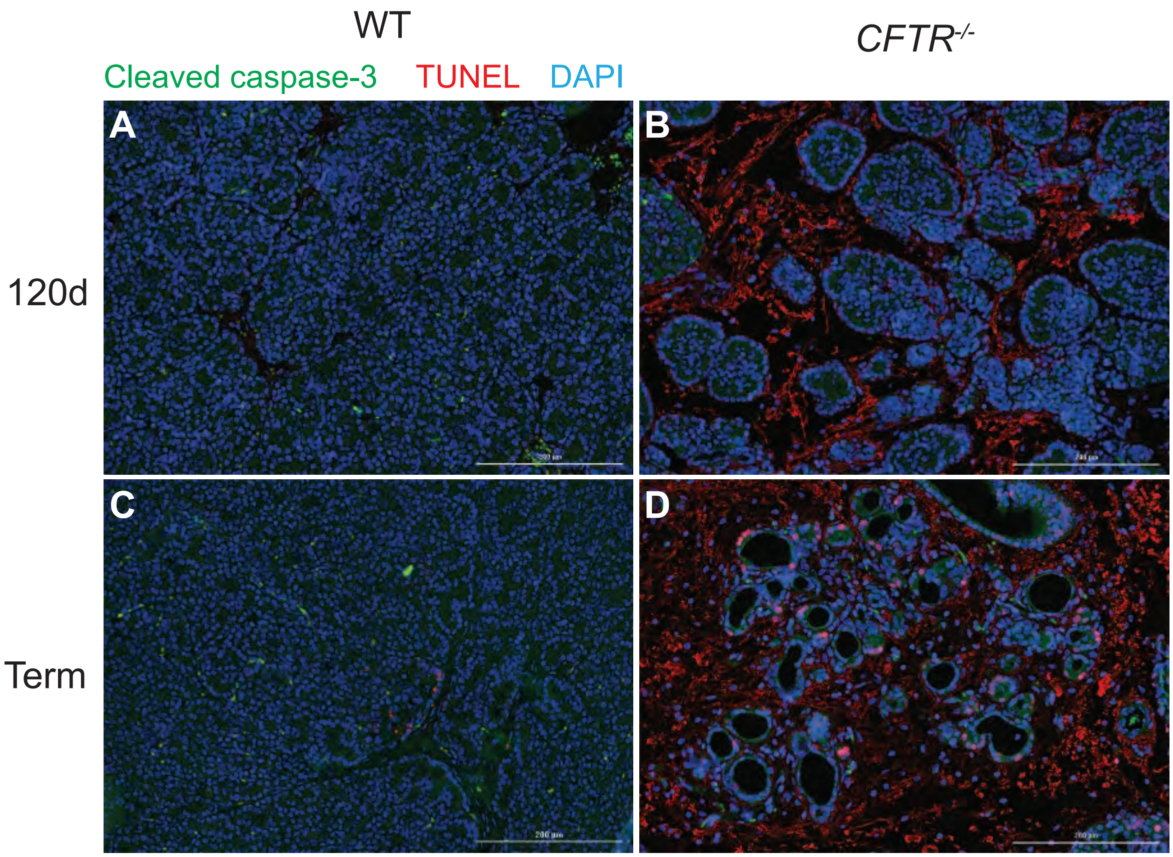

Figure S11. Elevated TUNEL staining in the CF pancreas at 120 days and term. Nuclei are counterstained with DAPI. Size bar = 200μM in all panels.

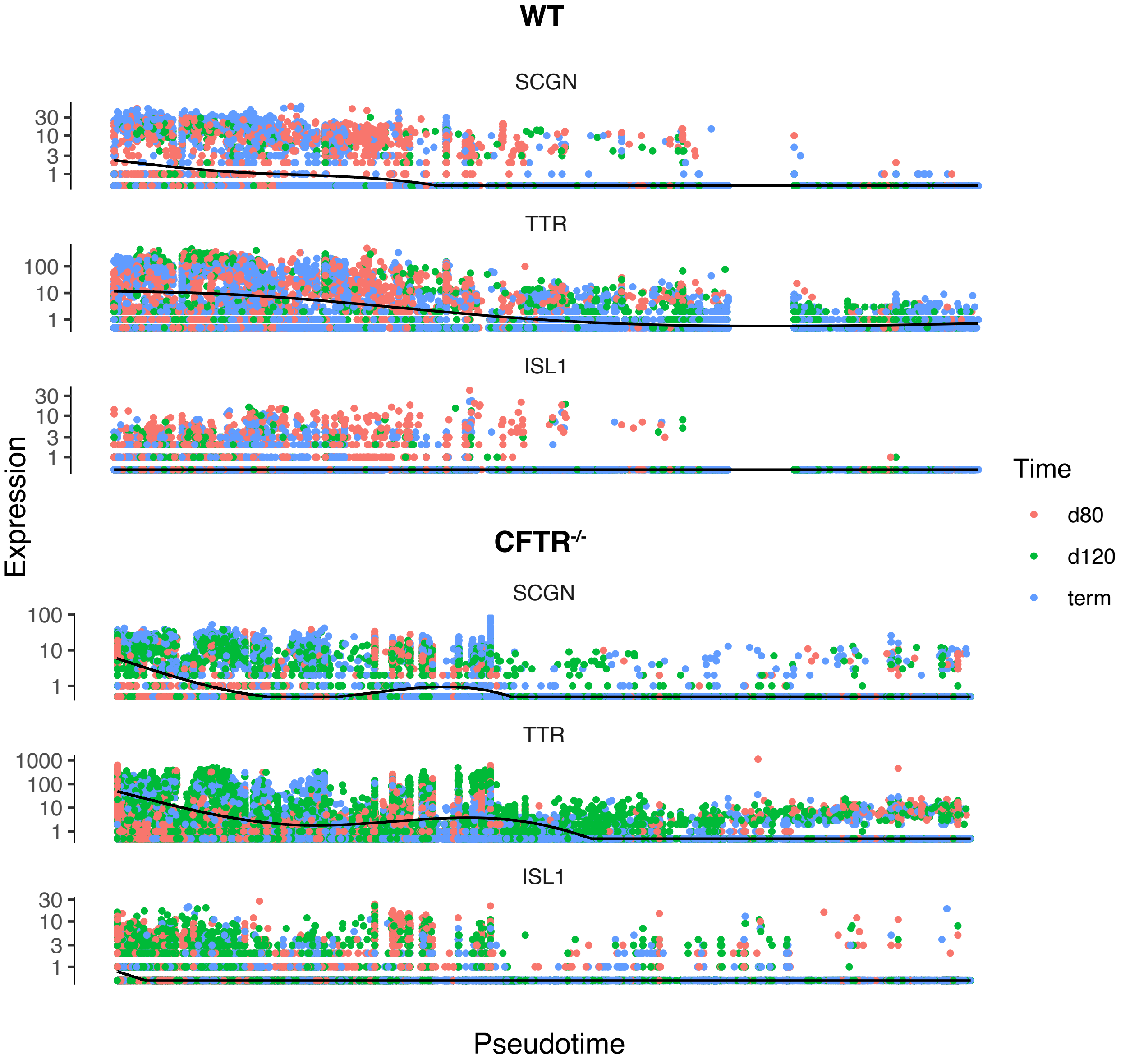

Figure S12. Pseudotime analysis of scRNA-seq data using Monocle 3 shows similar profiles for *SCGN*, *TTR* and *ISL1* in WT and *CFTR*$^{-/-}$ pancreatic endocrine cells.

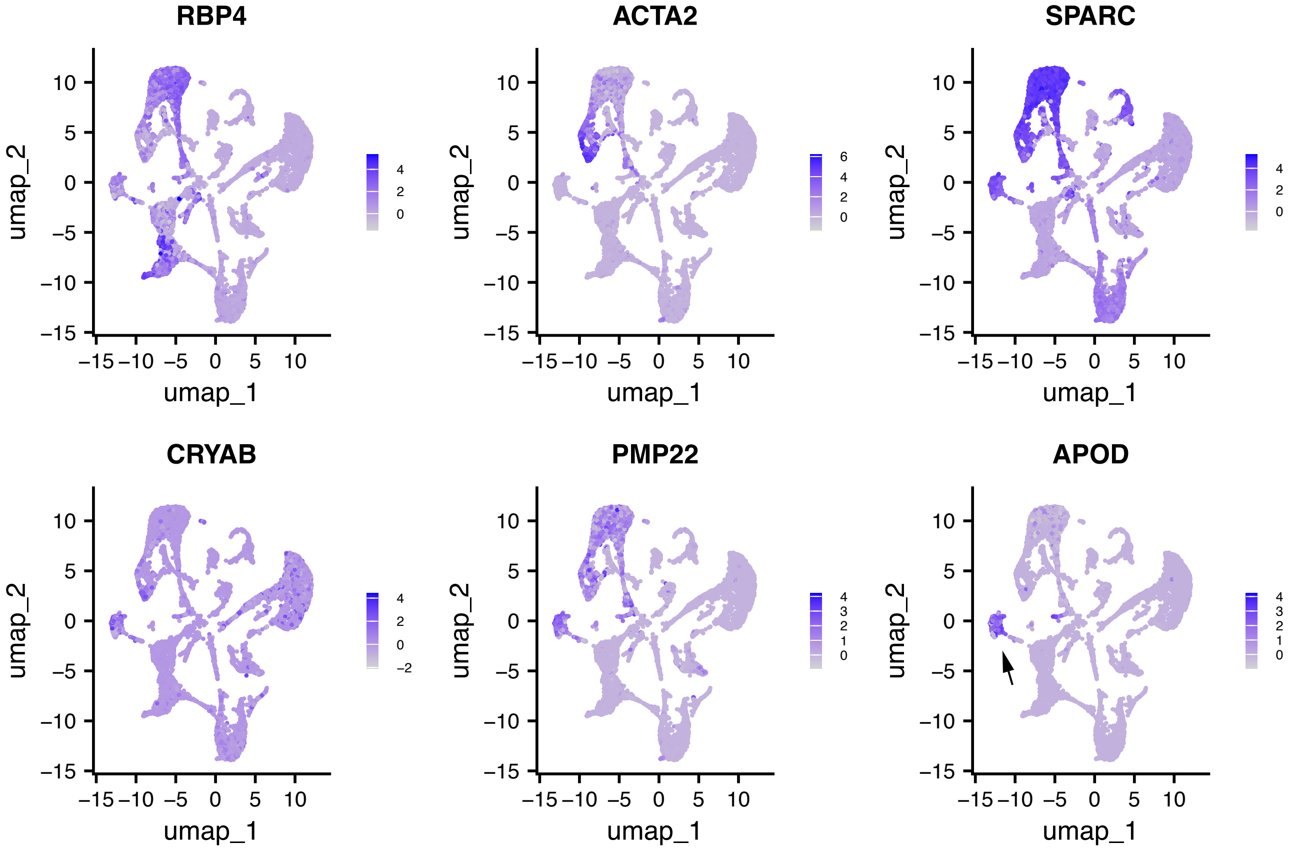

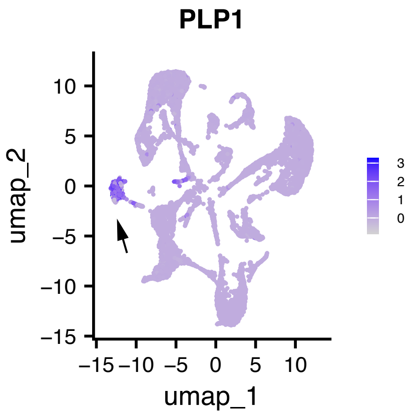

Figure S13A. Expression of marker genes for activated stellate cells and Schwann cells at 80 days gestation in merged data from WT and *CFTR*$^{-/-}$ pancreas. The unique cell cluster 6 is marked by the arrow.

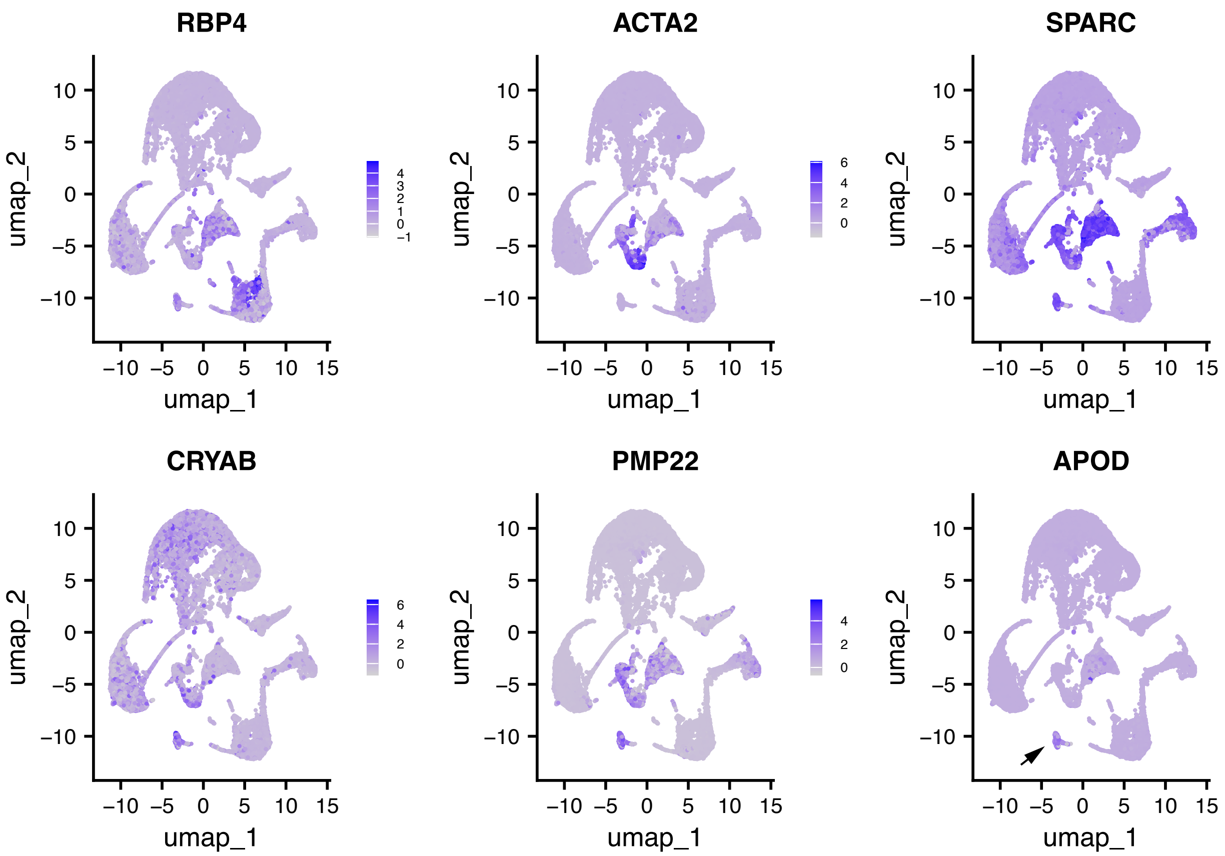

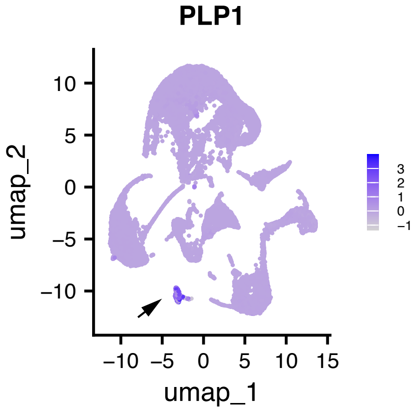

Figure S13B. Expression of marker genes for activated stellate cells and injured Schwann cells at 120 days gestation in merged data from WT and *CFTR*$^{-/-}$ pancreas. The unique cell cluster 8 is marked by the arrow.

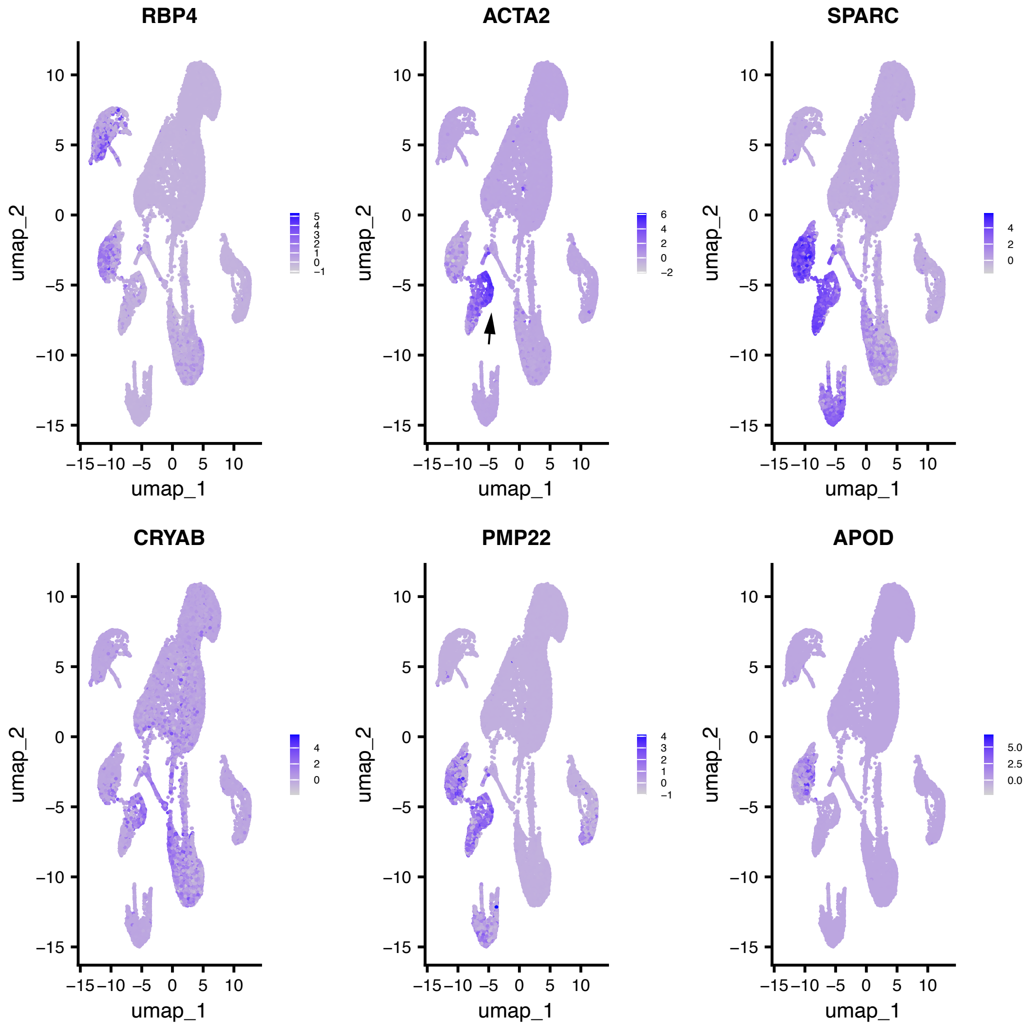

Figure S13C. Expression of marker genes for activated stellate cells and Schwann cells at term in merged data from WT and *CFTR*$^{-/-}$ pancreas. Activated stellate cells are marked by the arrow.
